# Effectiveness of Improvement Plans in Primary Care Practice Accreditation: A Clustered Randomized Trial

**DOI:** 10.1371/journal.pone.0114045

**Published:** 2014-12-02

**Authors:** Elvira Nouwens, Jan van Lieshout, Margriet Bouma, Jozé Braspenning, Michel Wensing

**Affiliations:** 1 Scientific Institute for Quality of Healthcare (IQ healthcare), Radboud University Medical Centre, Nijmegen, The Netherlands; 2 Dutch College of General Practitioners, Utrecht, The Netherlands; Hospital de Clínicas de Porto Alegre, Brazil

## Abstract

**Background:**

Accreditation of healthcare organizations is a widely used method to assess and improve quality of healthcare. Our aim was to determine the effectiveness of improvement plans in practice accreditation of primary care practices, focusing on cardiovascular risk management (CVRM).

**Method:**

A two-arm cluster randomized controlled trial with a block design was conducted with measurements at baseline and follow-up. Primary care practices allocated to the intervention group (n = 22) were instructed to focus improvement plans during the intervention period on CVRM, while practices in the control group (n = 23) could focus on any domain except on CVRM and diabetes mellitus. Primary outcomes were systolic blood pressure <140 mmHg, LDL cholesterol <2.5 mmol/l and prescription of antiplatelet drugs. Secondary outcomes were 17 indicators of CVRM and physician's perceived goal attainment for the chosen improvement project.

**Results:**

No effect was found on the primary outcomes. Blood pressure targets were reached in 39.8% of patients in the intervention and 38.7% of patients in the control group; cholesterol target levels were reached in 44.5% and 49.0% respectively; antiplatelet drugs were prescribed in 82.7% in both groups. Six secondary outcomes improved: smoking status, exercise control, diet control, registration of alcohol intake, measurement of waist circumference, and fasting glucose. Participants' perceived goal attainment was high in both arms: mean scores of 7.9 and 8.2 on the 10-point scale.

**Conclusions:**

The focus of improvement plans on CVRM in the practice accreditation program led to some improvements of CVRM, but not on the primary outcomes.

ClinicalTrials.gov NCT00791362

## Introduction

Accreditation of healthcare organizations is a widely used method to assess and improve the quality of healthcare. Most accreditation systems assess and rate the performance of organizations and service by evaluating their progress and appraising their compliance with standards, using mechanisms such as self-assessment surveys, data review and structured visits by surveyors [1]. Although the terms accreditation and certification are often used interchangeably, accreditation usually applies to healthcare organizations, while certification applies to practitioners and organizations [2]. In many countries accreditation is also emerging in primary care [3]. In the Netherlands, primary care practice accreditation is a voluntary activity comprising of an extensive audit, which covers clinical and organizational domains, followed by structured planning of improvements and formal review by an external assessor [4]. The program was initiated by the Dutch College of General Practitioners (DCGP) and is delivered by an independent organization (NPA). Improvement of professional performance and practice organization are prominent in the Dutch program [5]–[7].

Rigorous evaluations of the effectiveness of practice accreditation are rare [8]. Effects of accreditation on clinical performance, organizational processes and financial status are inconsistent and most studies focus on hospital care [9]. A study of practice accreditation in German primary care practices showed that it improved aspects of practice organization, but this study did not measure the effect on clinical processes or outcomes [10]. Given the role of audit and feedback in practice accreditation, research on this strategy may provide clues to the potential impact. A Cochrane review with 150 trials found that audit and feedback had a median effect of 4% improvement on aspects of professional performance, with substantial heterogeneity of effect sizes across studies. Audit and feedback combined with target setting and action planning, which is done in the Dutch practice accreditation, had 11% effect of measures of professional performance [11]. The Dutch practice accreditation model was an innovative approach of accreditation, because of its focus on learning and improving. In the Netherlands the majority of CVD patients receive necessary cardiovascular risk management in primary care practices [12]. In this paper we report on a study, which aimed to assess the effectiveness of improvement plans in practice accreditation of primary care practices, focused on cardiovascular risk management (CVRM).

## Methods

The protocol for this trial and supporting CONSORT checklist are available as supporting information; see Checklist S1 and Protocol S1.

### Trial design

The study design was a two-arm cluster randomized controlled trial with a block design, taking primary care practices as units of clustering, with measurements at baseline and at follow-up in independent samples of patients. The study protocol was published elsewhere [13]. The trial was registered at clinicaltrials.gov nr NCT00791362, http://www.clinicaltrials.gov/ct2/show/NCT00791362?term=NCT00791362&rank=1

### Ethical approval and informed consent

The Medical Ethics committee Arnhem-Nijmegen waived approval for this trial after assessing the study protocol (file number 2008/258). For the baseline-measurement mandatory information on indicators for patients with established CVD was used collected by practices on behalf of the practice accreditation program. At follow-up patients were requested informed consent in writing for permission to audit their medical records. The privacy of the participating patients was protected, and all data was coded and processed anonymously.

### Participants

#### Primary care practices

Primary care practices were recruited from practices in the Netherlands who had applied to start the practice accreditation program. After baseline data collection practices were randomized to study arms. Participating practices were randomized to a group which was instructed to improve CVRM (intervention arm) or to a group which was instructed to postpone improvement in CVRM or DM (control arm) until the intervention period was finished. Practices with a clear preference for a specific improvement plan were excluded from participation in the study.

All practices received a minimum of 4 hours support by outreach consultants for free.

Practices were recruited between September 2008 and April 2010. The date of receiving accreditation was the starting point of the intervention. Data concerning follow-up measurement were collected from February 2010 until May 2012, over the course of 12 months after the starting point of the intervention.

#### Patients

The study focused on patients with established atherosclerosis-related cardiovascular disease, as defined by prevailing clinical guidelines and recorded in patients' medical records [14], [15], including angina pectoris (K74), acute myocardial infarction (K75), other chronic ischemic heart diseases (K76), transient ischemic attack (K89), ischemic stroke (K90.3), peripheral arterial disease (K92.1) and aneurysma aortae (K99.1). Patients had to be in treatment for established CVD for a minimum period of 12 months. Patient selection from electronic medical records was based on corresponding diagnostic International Classification of Primary Care codes (ICPC-codes), an international classification system that is widely used in the Netherlands [16].

### The practice accreditation program

The practice accreditation program is a service, which has been offered since 2005. Practices have to comply to some minimum standards in order to be eligible for participation [17]. It is a comprehensive program including elements of clinical performance, practice organization and patient experiences. The program focuses strongly on chronic illness care, particularly diabetes mellitus (DM), asthma, COPD, and cardiovascular disease (CVD).

The practice accreditation program comprises, firstly, of a comprehensive audit (using validated performance indicators on a randomly selected sample of 40 patients per clinical domain) and written feedback to the practice, which covers a range of clinical domains (CVRM, DM, asthma and COPD), practice management, and patient experiences. The feedback, which consists of a comparison with benchmarks of other primary care practices, is discussed with a trained observer in a feedback consultation with the whole practice team and helps to identify substandard performance domains. The second obligatory component, the planning of improvements in the practice according to the principles of quality management, are based on this feedback. The practice team may chose to rely on a trained consultant to develop an improvement plan. Practices which perform the procedure as planned are all accredited, so accreditation does not imply that a certain minimum score on performance indicators has been obtained. In the practice accreditation program validated instruments are used: VIP [18], clinical indicators [19] and Europep [18]–[20]. Practices could claim an allowance for the costs of the accreditation program from health insurance companies. Furthermore they received a certification for the time period of one year which demonstrates (to the public) their involvement in continuous quality improvement. Every year the practice will be audited and every year new improvement plans have to be formulated which have to be approved by the auditor. The prolongation of the accreditation depends on having met the objectives of the improvement plans.

### Outcomes

Primary results were selected from the 20 quality indicators for established CVD [15], which were developed by the DCGP: the percentage of patients with known established CVD with systolic blood pressure below 140 mmHg, the percentage of patients with known established CVD with a LDL cholesterol level below 2.5 mmol/l, and the percentage of patients with known established CVD with a record that aspirin, an alternative anti-platelet therapy or an anti coagulant has been prescribed. Secondary outcomes consisted of the 17 remaining indicators and included: measurement of systolic blood pressure, measurement of LDL-cholesterol, prescription of statin, smoking status, patient is a smoker, stop smoking advice, measurement of Body Mass Index, Body Mass Index <25 kg/m^2^, measurement of waist circumference, fasting glucose measurement, influenza vaccination, registration of alcohol intake, control and advice for exercise and diet and comprehensive risk assessment. The indicators consist of process indicators, which give an indication of the progress of processes in an organization and outcome indicators, which give an indication of the outcome of care.

Medical data extraction was performed using a standardized procedure and documented for each included patient.

Another secondary outcome was the perceived goal attainment in the chosen improvement plans. This was documented in interviews with general practitioner or nurse on a likert-scale.

### Sample size

In the practices who voluntarily applied the practice accreditation program up to 2006 (n = 139) the following median values at practice level were found on indicators referring to patients with CVD: 53% for acceptable blood pressure levels; 36% for acceptable cholesterol levels; and 38% for use of anti coagulants (unpublished data, 2006). These data suggest that the scores on the primary outcomes are in the range of 36 to 53%, which imply that substantial improvement is possible in many practices. The proposed study was powered to detect a difference of 10% on all primary outcomes. We expected the practice accreditation program had an effect of 5% to 10% absolute change, which is the median value of effect sizes in a comprehensive review of 235 studies on quality improvement [7]. Other assumptions were a power  = 0.80, alpha  = 0.05, and ICC  = 0.05 [21]. Given the sample of 30 patients per practice per indicator, we aimed to include 31 practices in each group. Allowing for drop-out, we aimed at 35 practices in each group.

### Randomization

General practices were the unit of randomization. A computer list of random numbers was generated and used to randomly allocate practices to equally sized intervention group or control group by an independent statistician. This was done in a randomized block design in blocks of four practices based only on time period in order of enrolment.

### Blinding

General practitioners were aware of the allocated arm as the intervention consisted of making and implementing their own improvement plans. Data collectors were blinded to allocation. Blinding of patients was unnecessary as only medical records were assessed.

### Statistical methods

Descriptive data were analyzed using the SPSS 16.0 software package (Chicago, Illinois, USA). All indicators (all dichotomous measures) were included in a two-level logistic regression, taking into account the hierarchical structure of our study (patients nested within practices). In the logistic model covariates on the practice level that were taken into account included practice located in deprived area, availability of nurses for CVRM-related tasks and practice type (solo, duo, group). Patient's co-morbidity, age and sex were also included in the regression models. The analysis was performed in the SAS 9.2 package with procedure PROC GLIMMIX. We used a logistic regression model with a binomial distribution, a logit link function, a random intercept, and all other variables fixed.

Perceived goal attainment of participants was analyzed in a one-level regression model.

## Results

**Figure 1:**
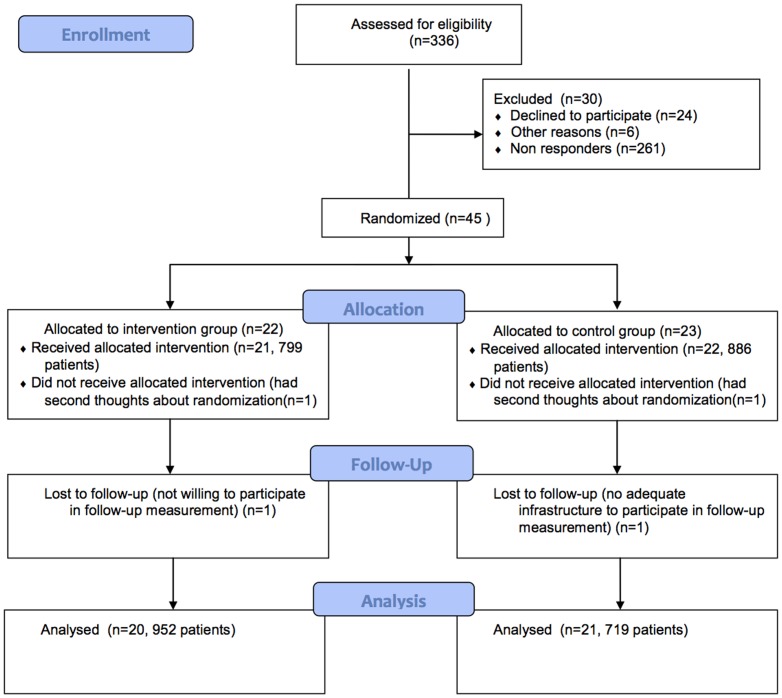
Flow Diagram.

336 Practices applied for the practice accreditation program in the recruitment period and were invited to participate in the study. 45 Practices were willing to participate in the study (Figure 1). A total of 22 practices was allocated to the intervention group en 23 practices to the control group. For follow-up measurement data on 20 practices were available in the intervention arm and data on 21 practices in the control arm.

Table 1 presents the characteristics of practices in the intervention and the control arm. In the intervention arm 57.1% of practices were solo practices, 19.0% were duo practices and 23.8% were group practices. For control arm practices this was 36.4%, 36.4% and 27.3% respectively. Of practices in the intervention group 6.3% participated in a care group with focus on CVRM, for control arm practices this was 35.0%. Table 2 presents characteristics of patients in the study population. At baseline 799 patients were included in the intervention group and 886 patients were included in the control group. At follow-up measurement 952 patients were included in the intervention group and 719 in the control group. In both study groups most common co-morbidity was diabetes and most common cardiovascular history was angina pectoris.

**Table 1:** Characteristics of Practice Population, Intervention vs. Control group.

	Intervention		Control	
	T0	T1	T0	T1
Number of practices	21	20	22	21
Number of patients	799	952	886	719
Practice Size (mean)	4417	4487	3559	3559
Solo practice	57.1%		36.4%	
Duo practice	19.0%		36.4%	
Group practice	23.8%		27.3%	
FTE FP	2.0 (SD 1.5)		1.7 (SD 0.8)	
FTE practice assistants	2.5 (SD 1.9)		2.1 (SD 1.3)	
FTE nurse practitioners	0.8 (SD 0.6)		0.7 (SD 0.5)	
FTE nurses	3.3 (SD 2.4)		2.8 (SD 1.7)	
Training practices	84.0%		60.0%	
Participation in care Group* with focus on CVRM	6.3%		35.0%	

*Regional organizations that have contracts with health insurers to coordinate CVRM-related care in a particular region with the objective to improve quality of care.

**Table 2:** Characteristics of Patient Population, Intervention versus Control group.

	Intervention		Control	
	T0 (n = 799)	T1 (n = 952)	T0 (n = 886)	T1 (n = 719)
Age (years)	69.41 (sd 11.65)	69.42 (sd 10.04)	68.88 (sd 12.22)	68.02 (sd 10.29
% Female	37.0	32.6	39.1	33.7
Diabetes (%)	23.2	16.9	25.2	22.8
COPD (%)	10.5	10.2	10.7	11.5
Astma (%)	5.6	6.4	4.0	6.5
		
Angina Pectoris	37.8	33.3	35.7	31.8
Myocardial Infarction	31.1	31.1	27.7	28.1
Other chronic ischemic heart diseases	9.4	10.7	12.1	17.1
TIA	14.3	14.8	15.2	13.6
Ischemic stroke	5.0	10.8	8.4	8.9
Peripheral arterial disease, claudicatio intermittens	8.8	11.0	14.3	13.8
Aneurysma Aortae	4.8	5.3	5.0	7.9

### Primary outcomes

None of the primary outcomes showed significant improvements (Table 3). Patients with a systolic blood pressure <140 mmHg decreased in the study arm (from 50.5% to 39.8%) as well as the control arm (from 50.6% to 38.7%). Patients with LDL cholesterol <2.5 mmol/L hardly changed. Patients with a prescription of antiplatelet drugs decreased in the intervention group from 84.3% to 82.7% and in the control group from 84.9% to 82.7%.

**Table 3:** Record of indicators for cardiovascular risk management in electronic medical records.

		Intervention group			Control group			
Type of indicator		Baseline T0 (n = 799)	Follow-up T1 (n = 952)	Change %	Baseline T0 (n = 886)	Follow-up T1 (n = 719)	Change %	Between Group change %
Outcome	Systolic blood pressure <140 mmHg	297/588 (50.5)	283/712 (39.8)	−10.7	342/676 (50.6)	225/581 (38.7)	−11.9	1.2
Process	Systolic blood pressure measured	588/796 (73.9)	712/948 (75.1)	+1.2	676/886 (76.3)	581/716 (81.1)	+4.8	−3.6
Outcome	LDL cholesterol <2.5 mmol/l	174/382 (45.6)	232/521 (44.5)	−1.1	215/448 (48)	217/443 (49)	+1.0	−2.1
Process	LDL cholesterol measured	384/793 (48.4)	521/937 (55.6)	+7.2	448/884 (50.7)	443/716 (61.9)	+11.2	−3.0
Process	Patients with LDL cholesterol ≥2.5 mmol/l with statin prescription	137/207 (66.2)	200/289 (69.2)	+3.0	146/233 (62.7)	176/225 (78.2)	+15.5	−12.5
Process	Prescription antiplatelet drugs	671/796 (84.3)	787/952 (82.7)	−1.6	751/885 (84.9)	593/717 (82.7)	−2.2	−0.6
Process	Smoking status	293/796 (36.8)	609/951 (64)	+27.2	436/882 (49.4)	457/719 (63.6)	+14.2***	13.0
Outcome	Patient is a smoker	93/292 (31.9)	134/609 (22)	−9.9	136/436 (31.2)	123/457 (26.9)	−4.3	5.6
Process	Stop smoking advice	58/93 (62.4)	69/133 (51.9)	−10.5	67/134 (50)	65/118 (55.1)	+5.1	−15.6
Process	Exercise control	220/798 (27.6)	357/951 (37.5)	+9.9	264/885 (29.8)	234/715 (32.7)	+2.9***	7.0
Process	Advice physical activity	155/798 (19.4)	198/951 (20.8)	+1.4	191/884 (21.6)	182/714 (25.5)	+3.9	−2.5
Process	Diet control	191/799 (23.9)	261/949 (27.5)	+3.6	237/886 (26.8)	185/715 (25.9)	−0.9*	4.5
Process	Counseling about diet	196/799 (24.5)	266/952 (27.9)	+3.4	224/883 (25.6)	230/716 (32.1)	+6.5	−3.1
Process	Registration of alcohol intake	197/797 (24.7)	383/941 (40.7)	+16.0	277/885 (31.3)	266/711 (37.4)	+6.1***	9.9
Process	Waist circumference measured	87/788 (11)	158/938 (16.8)	+5.8	125/873 (14.3)	140/705 (19.9)	+5.6*	0.2
Process	Fasting glucose measured	501/799 (62.7)	687/944 (72.8)	+10.1	621/881 (70.5)	516/714 (72.3)	+1.8*	8.3
Process	BMI measured	217/797 (27.2)	362/921 (39.3)	+12.1	241/882 (27.3)	278/695 (40)	+12.7	−0.6
Outcome	BMI <25 kg/m^2^	33/217 (15.2)	66/362 (18.2)	+3.0	46/241 (19.1)	45/278 (16.2)	−2.9	5.9
Process	Influenza vaccination	609/799 (76.2)	475/758 (62.7)	−13.5	669/884 (75.7)	520/634 (82)	+6.3	−19.8
Process	Comprehensive risk assessment 1	32/799 (4)	63/952 (6.6)	+2.6	51/886 (5.8)	62/719 (8.6)	+2.8	−0.2

*P-value <0.05 (difference in change corrected for patients nested in practices).

***P-value <0.001 (difference in change corrected for patients nested in practices).

1 positive score when there is a record of: blood pressure, BMI, waist circumference, fasting glucose measurement, LDL cholesterol measurement, smoking behavior, alcohol intake, advice and control of diet and physical exercise in the past 12 months.

### Secondary outcomes

Of the 17 secondary outcomes, six showed significant improvements as a result of the intervention. These were, patients with known smoking status (27.2% and 14.2% change respectively; p = <0.0001); registration of physical exercise (9.9% and 2.9% change respectively; p = 0.0042); registration of diet control (3.6% and 0.9% change respectively; p = 0.0258); registration of alcohol intake (16.0% and 6.1% change respectively; p = 0.0007); measurement of waist circumference (5.8% and 5.6% change respectively; p = 0.0346) and measurement of fasting glucose (10.1% and 1.8% change respectively; p = 0.0360). The other 11 secondary outcomes did not show significant changes.

### Perceived goal attainment

The objectives of the chosen improvement plans on CVRM mainly concerned the establishment of a CVRM-consultation hour, the identification of patients eligible for CVRM and the improvement of registration in electronic medical records. The participants' perceived goal attainment on plans concerning chronic care management was documented for 30 practices. The findings suggest that goals were largely perceived to be met (Table 4). In the intervention arm the mean score for goal achievement was 7.9 (SD 1.2) and in the control group the mean score was 8.2 (SD = 1.2). There was no significant difference between the study arms (p = 0.45) which implies CVRM related goals were achieved to the same extent as goals focusing on other domains of chronic care.

**Table 4:** Perceived goal attainment on plans concerning chronic care management^1^.

	Intervention a (N = 14)	Control b (N = 16)
Min	6	6
Max	10	10
Mean	7.9	8.2
SD	1.2	1.2

1 Measured on a Likert scale (1–10).

a plans concerning CVRM.

b plans concerning chronic care management other than CVRM.

## Discussion

The Dutch accreditation program for primary care practices is strongly focused on learning and improving healthcare delivery, using a comprehensive audit and feedback procedure that is largely focused on the management of chronic diseases. We found that this program improved some aspects of professional performance concerning CVRM in the practices who focused their improvement plans on CVRM, but not on the primary outcomes. The participants largely perceived to achieve chosen goals of their improvement projects.

Although accreditation schemes have been evaluated in observational studies, this is one of the first controlled evaluations of this method to enhance quality of healthcare. A notable exception is a controlled study in German primary care practices [10], which also reported positive effects, however, this German accreditation program focused on organizational domains rather than clinical processes. If we compare our primary outcomes with the results of trials of audit and feedback (a key component of the Dutch practice accreditation), we found effects at lower end of the range of effect sizes. The effects on a few secondary outcomes were only slightly higher than other studies of audit and feedback, combined with target setting and action planning, have found. So, this study did not provide evidence that the context of practice accreditation had an added value to the effectiveness of the audit and feedback.

A possible explanation for the lack of stronger effects is the impact of patient related factors on the outcomes, such as poor compliance with treatment [22] and patients' comorbidity. Furthermore, a substantial part of practices in the intervention arm were solo practices. Group practices might have more defined processes to address quality issues.

In the follow-up measurement the number of patients with diabetes decreased. A smaller contribution of this otherwise relatively well treated sub group [23] will lead to lower overall scores.

A number of secondary outcomes improved more in the intervention arm. Assuming participating in the accreditation program induces better monitoring of patients and improvement of registration behavior in general, we would expect all secondary outcomes to improve and not only the six outcomes as demonstrated in this study.

Practices in the control arm also showed improvements on the measures of CVRM quality at follow up. This might be explained by increased attention on CVRM in integrated care groups, increased awareness for quality of care in general in addition to improvement plans and furthermore increased awareness of registration behavior in general when participating in an accreditation program.

### Strengths and weakness of the study

To our knowledge this is one of the first trials of an accreditation program in primary care. The performance indicators in the program were carefully developed [24], [25]. Data in this study were manually collected from electronic medical support systems. The sample size calculated was 30 patients per practice. However, practices participating in the practice accreditation were required to collect data on 40 patients which gives more body of evidence to the baseline-measurement. In the analysis baseline-measurements were included in the model which amplifies the power and therefore compensates for the calculated number of 35 clusters per group that was not achieved.

Follow-up of the same cohort of patients would have been more efficient but was not feasible. We measured aspects of clinical process and outcomes on patients as an indicator of change in clinicians, who remained the same throughout the study. The different samples were taken into account in the data-analysis approach, resulting in somewhat reduced accuracy compared to following up the same cohort of patients.

The control group in this study also showed improvements. This could be the effect of contamination as practices in the control group also participated in the Dutch accreditation program. A different study design might have demonstrated a larger effect, but this was not feasible. Another limitation of the study is the risk of selection bias in the follow-up measurement as patients had to give informed consent for data collection from their electronic medical record. Selection bias may also be the effect of the fact that randomization only occurred in order of time of enrolment due to feasibility problems.

Randomization determined the focus on CVRM for the improvement plans in the first year of the cycle, ideally the outcome of feedback determines the focus of the plans. Furthermore this might explain why invited practices declined to participate. In this study practices could establish their own goals for improvement plans without limitations or guidance. If plans would be more focused on improvement of outcome measures, the effects might have been larger.

It was not feasible to assess outcomes on patient level such as death, myocardial infarction or stroke, however it would have been interesting to examine if the accreditation program is of influence on these outcomes.

We have failed to mention the covariates included in this study with registration of this trials. However, the covariates were discussed in the published studyprotocol [13].

### Generalizability

General practices in this study all voluntarily applied for the practice accreditation program. This could imply that practices included in this study have a more than average affinity with quality of care and have higher baseline measurements and therefore have less to improve. Furthermore the practice accreditation program was initiated in 2005, practices included in this study are the early adaptors [26] among general practices in the Netherlands, especially taking into account the program is voluntary, and for that reason more eager to initiate improvement. A substantial number of practices in our study are training practices. Of these practices it is to be expected they are more open to innovations. On the other hand they may have felt pressure to participate.

The results of our study can be compared to a large observational study in European primary care (EPA-Cardio), which provided data on CHD on the basis of validated quality indicators in eight European countries, including the Netherlands [27]. In Dutch practices in EPA-cardio 28.9% of patients had a systolic blood pressure below 140 mmHg, which is lower than in our study at both baseline and follow-up measurement. In addition, 43.0% of patients in EPA-cardio had a LDL cholesterol level below 2.5 mmol/l which was comparable to our sample. Anti-platelet drugs were prescribed in 82.8% of patients which was also comparable to the results of our study. So, accrediting practices in our study are comparable to other practices in the Netherlands.

### Implications

The Dutch accreditation program for primary care practices is a method which encourages practice teams to use a planned and cyclic approach to learning and improving their performance. It intends to stimulate improvement in organizational and clinical domains, focusing largely on chronic illness care. This study showed there was ample room for improvement on all aspects of CVRM, which implies the legitimacy of the Dutch accreditation program. The accreditation program stimulates team collaboration [28], transparency of performance, and shared responsibility for delivering the best possible primary care. Although the primary outcomes did not show improvements, participating practices in our study perceived to achieve chosen goals for improvement projects to a large extent. Effects might be larger when this study would be repeated in the second or third year of the accreditation cycle when organizational aspects are improved and practices can focus more on the improvement of outcome measurements [29].

We believe it is too early to conclude that the accreditation program is not effective, because it includes a number of well established methods and principals of behavior change. To obtain more substantial improvements, goals in the improvement plans should be formulated related to outcome measurements. Furthermore, additional interventions may be required, such as financial incentives for practices with high performance or public reporting on quality scores. It is unlikely that these methods will be ‘magic bullets’ for improving healthcare delivery, but they may help to optimize the effectiveness of the program.

## Supporting Information

Checklist S1 **CONSORT Checklist.**
(DOC)

Protocol S1 **Trial Protocol.**
(PDF)

## References

[1] Braithwaite J, Westbrook J, Johnston B, Clark S, Brandon M, et al (2011) Strengthening organizational performance through accreditation research-a framework for twelve interrelated studies: the ACCREDIT project study protocol. BMC Res Notes 4:390. 21981910 10.1186/1756-0500-4-390 PMC3199265

[2] Alkhenizan A, Shaw C. (2011) Impact of accreditation on the quality of healthcare services: a systematic review of the literature. Ann Saudi Med 31(4):407–416. 21808119 10.4103/0256-4947.83204 PMC3156520

[3] Lester HE, Eriksson T, Dijkstra R, Martinson K, Tomasik T, et al (2012) Practice accreditation: the European perspective. Br J Gen Pract 62(598):e390–e392. 22546601 10.3399/bjgp12X641627 PMC3338063

[4] Buetow SA, Wellingham J. (2003) Accreditation of general practices: challenges and lessons. Qual Saf Health Care 12(2):129–135. 12679510 10.1136/qhc.12.2.129 PMC1743687

[5] Frijling B, Hulscher ME, van Leest LA, Braspenning JC, Van den Hoogen, et al (2003) Multifaceted support to improve preventive cardiovascular care: a nationwide, controlled trial in general practice. Br J Gen Pract 53(497):934–941. 14960217 PMC1314746

[6] Frijling BD, Lobo CM, Hulscher ME, Akkermans RP, van Drenth BB, et al (2003) Intensive support to improve clinical decision making in cardiovascular care: a randomised controlled trial in general practice. Qual Saf Health Care 12(3):181–187. 12792007 10.1136/qhc.12.3.181 PMC1743704

[7] Grimshaw JM, Thomas RE, MacLennan G, Fraser C, Ramsay CR, et al (2004) Effectiveness and efficiency of guideline dissemination and implementation strategies. Health Technol Assess 8(6):iii–72. 10.3310/hta8060 14960256

[8] Greenfield D, Braithwaite J. (2009) Developing the evidence base for accreditation of healthcare organisations: a call for transparency and innovation. Qual Saf Health Care 18(3):162–163. 19467994 10.1136/qshc.2009.032359

[9] Greenfield D, Braithwaite J. (2008) Health sector accreditation research: a systematic review. Int J Qual Health Care 20(3):172–183. 18339666 10.1093/intqhc/mzn005

[10] Szecsenyi J, Campbell S, Broge B, Laux G, Willms S, et al (2011) Effectiveness of a quality-improvement program in improving management of primary care practices. CMAJ 183(18):E1326–E1333. 22043000 10.1503/cmaj.110412 PMC3255110

[11] Ivers N, Jamtvedt G, Flottorp S, Young JM, Odgaard-Jensen J, et al (2012) Audit and feedback: effects on professional practice and healthcare outcomes. Cochrane Database Syst Rev 6:CD000259. 10.1002/14651858.CD000259.pub3 PMC11338587 22696318

[12] In 't Veld CJ, Grol RP. (2007) Practice guidelines and accreditation: highlights from 50 years of quality management by the Dutch College of General Practitioners. Ned Tijdschr Geneeskd 151(52):2916–2919. 18257441

[13] Nouwens E, Van Lieshout J, Adang E, Bouma M, Braspenning J, et al (2012) Effectiveness and efficiency of a practice accreditation program on cardiovascular risk management in primary care: study protocol of a clustered randomized trial. Implement Sci 7:94. 23035760 10.1186/1748-5908-7-94 PMC3533965

[14] **NHG-Standaard Cardiovasculair risicomanagement** (2006) Houten: Bohn Stafleu van Loghum.

[15] Van Althuis T (2008) Overzicht en definitie van indicatoren voor cardiovasculair risicomanagement bij patiënten met bekende hart- en vaatziekten in de huisartsenzorg. NHG.

[16] Soler JK, Okkes I, Wood M, Lamberts H. (2008) The coming of age of ICPC: celebrating the 21st birthday of the International Classification of Primary Care. Fam Pract 25(4):312–317. 18562335 10.1093/fampra/cmn028

[17] Van den Hombergh P, Schalk-Soekar S, Kramer A, Bottema B, Campbell S, et al (2013) Are family practice trainers and their host practices any better? Comparing practice trainers and non-trainers and their practices. BMC Fam Pract 14:23. 23433175 10.1186/1471-2296-14-23 PMC3598999

[18] Van den Hombergh P (1998) Practice visits. Assessing and improving management in general practice. Nijmegen: KUN.

[19] Van Doorn A, Kirschner K, Bouma M, Burgers J, Braspenning J, et al (2010) Evaluatie van het onderdeel medisch handelen van de accreditering. Vier klinimetrische criteria. Huisarts Wet 53(3):141–146.

[20] Grol R, Wensing M, Mainz J, Jung HP, Ferreira P, et al (2000) Patients in Europe evaluate general practice care: an international comparison. Br J Gen Pract 50(460):882–7. 11141874 PMC1313852

[21] Campbell MK, Fayers PM, Grimshaw JM. (2005) Determinants of the intracluster correlation coefficient in cluster randomized trials: the case of implementation research. Clin Trials 2(2):99–107. 16279131 10.1191/1740774505cn071oa

[22] Furthauer J, Flamm M, Sonnichsen A. (2013) Patient and physician related factors of adherence to evidence based guidelines in diabetes mellitus type 2, cardiovascular disease and prevention: a cross sectional study. BMC Fam Pract 14:47. 23557543 10.1186/1471-2296-14-47 PMC3623850

[23] Nouwens E, Van Lieshout J, Wensing M. (2012) Comorbidity complicates cardiovascular treatment: is diabetes the exception? Neth J Med 70(7):298–305. 22961822

[24] Braspenning J, Pijnenborg L, In 't Veld CJ, Grol R (2005) Werken aan kwaliteit in de huisartsenpraktijk. Indicatoren gebaseerd op de NHG-Standaarden. Houten: Bohn Stafleu Van Loghum.

[25] Engels Y (2005) Assessing and improving management in primary care practice in the Netherlands and in Europe. Nijmegen: UMC St Radboud.

[26] Rogers EM (1983) Diffusion of innovations. New York: The Free Press.

[27] Van Lieshout J, Grol R, Campbell S, Falcoff H, Capell EF, et al (2012) Cardiovascular risk management in patients with coronary heart disease in primary care: variation across countries and practices. An observational study based on quality indicators. BMC Fam Pract 13:96. 23035928 10.1186/1471-2296-13-96 PMC3515459

[28] Van Doorn-Klomberg AL, Kirschner K, Bouma M, In 't Veld CJ, Wensing M, et al (2011) Ervaringen met de NHG-Praktijkaccreditering. Huisarts Wet 54(7):360–365.

[29] Smeel I, Meulepas M, Meulemans C, Reus I, Klomp M. (2012) Eerste ervaringen met COPD ketenzorg. Huisarts Wet 55(5):194–198.

