# Supplementary material for: Effectiveness of Improvement Plans in Primary Care Practice Accreditation: A Clustered Randomized Trial
Source: PLoS One. 2014 Dec 2;9(12):e114045. doi: 10.1371/journal.pone.0114045 (PMC4252098; doi:10.1371/journal.pone.0114045)
Supplement: Protocol S1 — Trial Protocol. (PDF) [file pone.0114045.s002.pdf]

**DEFINITIEF**

# Algemene gegevens / General Information

### Programma / Programme

DoelmatigheidsOnderzoek: deelprogramma Implementatie

### Subsidieronde / Subsidy round

IMP - round 08 - grant application

### Projecttitel / Project title

Effects and costs of a national continuous improvement programme on cardiovascular diseases in primary care

### Aanvrager / Applicant

Dr. M Wensing

*Functie / Position:* | *Opleiding / Education:*

*Studierichting / Subject:*

*T:* 024-3614227 | *F:* 024-3540166 | *E:* m.wensing@kwazo.umcn.nl

Universitair Medisch Centrum St. Radboud

Kwaliteit van Zorg/VOHA

114 KWAZO

Postbus 9101

6500 HB NIJMEGEN

Nederland

### Projectleden / Project members

Dr. M Wensing (Projectleider en penvoerder)

*Functie / Position:* Senior Lecturer (UHD) | *Opleiding / Education:*

*Studierichting / Subject:*

*T:* 024 3615305 | *F:* | *E:* M.Wensing@kwazo.umcn.nl

Universitair Medisch Centrum St. Radboud

Kwaliteit van Zorg (WOK)

117 KWAZO

Postbus 9101

6500 HB NIJMEGEN

Nederland

Prof. dr. D.J. Ruiter (Bestuurlijk verantwoordelijke)

*Functie / Position:* interim chairman Hospital Board | *Opleiding / Education:*

*Studierichting / Subject:*

*T:* 024-3619603 | *F:* | *E:* doelmatigheidsonderzoek@medzaken.umcn.nl

Universitair Medisch Centrum St. Radboud

Raad van Bestuur

Postbus 9101

6500 HB NIJMEGEN

Nederland

Dr. Adang (Begeleider)
*Functie / Position:* Senior Researcher Fellow (HTA) | *Opleiding / Education:*
*Studierichting / Subject:*
*T:* 024-3601387 | *F:* | *E:*
Universitair Medisch Centrum St. Radboud
Medical Technology Assessment

Postbus 9101
6500 HB NIJMEGEN
Nederland

Ir. R Akkermans (Uitvoerder)
*Functie / Position:* Methodologist (quantitative analysis) | *Opleiding / Education:*
*Studierichting / Subject:*
*T:* 024-3615305 | *F:* | *E:*
Universitair Medisch Centrum St. Radboud
Kwaliteit van Zorg

Postbus 9101
6500 HB NIJMEGEN
Nederland

Dr. M Bouma (Begeleider)
*Functie / Position:* Senior Staff Member (practice accreditation) | *Opleiding / Education:*
*Studierichting / Subject:*
*T:* 030-1111111 | *F:* | *E:*
Nederlands Huisartsen Genootschap
Implementatie

Postbus 3231
3502 GE UTRECHT
Nederland

Dr. Braspenning (Mede aanvrager)
*Functie / Position:* Senior Research Felllow (quality indicators) | *Opleiding / Education:*
*Studierichting / Subject:*
*T:* 024-3615305 | *F:* | *E:*
Universitair Medisch Centrum St. Radboud
Kwaliteit van Zorg (WOK)
117 KWAZO
Postbus 9101
6500 HB NIJMEGEN
Nederland

**DEFINITIEF**

Prof. dr. Grol (Promotor)
*Functie / Position:* Professor, head of department | *Opleiding / Education:*
*Studierichting / Subject:*
*T:* 024-3615305 | *F:* | *E:*
Universitair Medisch Centrum St. Radboud
Kwaliteit van Zorg (WOK)
117 KWAZO
Postbus 9101
6500 HB NIJMEGEN
Nederland

Drs. C In 't Veld (Mede projectleider)
*Functie / Position:* Director NHG-department Implementation | *Opleiding / Education:*
*Studierichting / Subject:*
*T:* 030 0000000 | *F:* | *E:*
Nederlands Huisartsen Genootschap

Postbus 3231
3502 GE UTRECHT
Nederland

Drs. J van Lieshout (Hoofdonderzoeker)
*Functie / Position:* Clinical Research Fellow (GP) | *Opleiding / Education:*
*Studierichting / Subject:*
*T:* 024-3615305 | *F:* | *E:*
Universitair Medisch Centrum St. Radboud
Kwaliteit van Zorg (WOK)
117 KWAZO
Postbus 9101
6500 HB NIJMEGEN
Nederland

Dr. Van der Weijden (Begeleider)
*Functie / Position:* Senior Lecturer (UHD) | *Opleiding / Education:*
*Studierichting / Subject:*
*T:* 043-3882877 | *F:* | *E:*
Universiteit Maastricht
Faculteit der Geneeskunde
Capaciteitsgroep Huisartsgeneeskunde
Postbus 616
6200 MD MAASTRICHT
Nederland

**Samenwerking / Collaboration**

**DEFINITIEF**

## Projectgegevens / Project information

**Datum indienen (via ProjectNet) / Date of application**

20-07-2007 11:17

**Aandachtsgebieden / Focus**

*Themes:*

Implementatiestrategieën en -processen ;

*Themes HTA methodology:*

Evaluation research of aspects of organisation;

**Projecttype / Project type**

Onderzoeksproject

**Samenvatting / Summary**

Objective: This study aims to determine the effectiveness and efficiency of a national accreditation and improvement programme for general practice, focusing on patients with established cardiovascular diseases. An evidence-based multidisciplinary guideline on cardiovascular risk management was published late 2005. It contains a number of new recommendations for clinical treatment, particularly regarding cut off levels for treatment (e.g. LDL<2,5 and SBP<140) and choice of medication, which are difficult to implement in patient care.

Design: Cluster randomised trial with a block design. All practices start with the accreditation procedure. Intervention group practices are requested to focus their first improvement plans on cardiovascular disease. Control group practices are requested to focus their first improvement plans on other domains. Measurements at baseline are based on the standard audit in the accreditation procedure. Follow-up measurements are done 18 months later.

Data: Primary and secondary outcomes are based on the indicators in the accreditation procedure. In addition, data is collected at follow-up on perceived goal attainment (for improvement objectives chosen by the practice, in the intervention group only), resource use (needed for the economic evaluation), practice characteristics (potential confounders in the analysis of effectiveness), and perceived unintended consequences (process evaluation by telephone interviews with physicians).

Interventions to be implemented: pharmacological treatment, life style advice, systematic monitoring, and referral regarding cardiovascular risk management, as described by recently updated national multidisciplinary evidence-based NHG / CBO guidelines.

Implementation strategy: The national programme for accreditation and improvement of general practice. This procedure consists of a package of activities, including audit, feedback, improvement plans, and follow-up. It is partly based on new theories on quality improvement, including the use of market forces and pressure for accountability. For the purpose of the study, the intervention group will be requested to focus their first improvement plans on cardiovascular diseases. The control group is requested to focus their first improvement plans on other domains and to provide cardiovascular care as usual.

Outcome measures: Primary outcomes are percentages of patients with CVD who have acceptable systolic blood pressure and cholesterol levels (quantified according to the indicators in the accreditation) and who use aspirine or alternatives. Secondary measures include clinical and organisational indicators of quality of cardiovascular care, such as percentages of patients with cardiovascular disease whose risk

factors were assessed and who received specific medication.

Sample size calculation: The study is powered to detect a difference between 55% and 65% score on performance indicators. A total of 70 practices (35 per study group) is included, which each provide 30 patients per indicator.

Economic evaluation: Incremental cost effectiveness ratio's are determined of the implementation strategy compared to no implementation. The analysis will take a societal perspective and a time horizon of the observed period as well as a hypothetical 10 years period (using modelling). Uncertainty related to the estimations is examined with sensitivity analyses and bootstrapping. The long term economic evaluation is based on Markov modelling.

Time schedule: Practices recruitment and accreditation procedure (month 1-12); Follow-up measurement (month 18-30); Data-analysis and reporting (month 30-36).

**Trefwoorden / Keywords**

Institutional accreditation, professional certification, primary care, cardiovascular illness

## Inhoud / Content

### Probleemstelling / Problem definition

Healthcare problem.
The proposed project focuses on patients with established cardiovascular diseases (CVD) in primary care. The most relevant conditions are coronary heart disease (angina pectoris, myocardial infarction, heart surgery), transient ischaemetic attack and cerebrovascular accidents, and peripheral vascular disease. Most patients are middle-aged or older, and have more conditions for CVD simulaniously (multimorbidity). More men than women are affected, but CVD is rising in women. Etnicity is not clearly related to CVD prevalence. A completely revised guideline on cardiovascular risk management was published by NHG late 2005 and a slightly revised version of this was published in 2006 as a multidisciplinary CBO guideline [1]. Other NHG guidelines on CVD were updated in recent years (AP in 2004, MI in 2005, TIA in 2004, CVA in 2004, PVD in 2003). Shared care guidelines (LTA) are available for CVA/TIA, MI aftercare, and ACS. The new set of guidelines on CVD describe the clinical interventions to be implemented in patient care in this project. They contain important changes in recommendations, such as different cut-off levels (e.g. LDL<2,5 mmol/l and SBP<140 mmHg) and higher treatment targets for clinical intervention.

Effectiveness of interventions.
The evidence base of clinical interventions in CVD is strong, particularly in patients with established CVD. The guidelines on cardiovascular risk management [1] are based on explicit prediction of cardiovascular adverse events and on efficiency considerations regarding preventive intervention. Life style advice is targetted at stop smoking, physical exercise, healthy diet, weight reduction, moderate alcohol use. Pharmacological treatment comprises of cholesterol lowering medication, antihypertensives, antithrombolitic medication, and other disease specific medication. A GP can provide advice and treatment or refer to practice nurse, dietician, exercise programmes, or self-management programmes. Pharmacological treatment and life style change can reduce the risk for cardiovascular events by 20% or more (relative risk reduction) [1]. The absolute risk reduction depends on the baseline level of risk.

Implementation problem.
A wide range of activities is targetted at primary care to improve cardiovascular care, such as

educational materials provided by the Dutch College of GPs (NHG); projects of regional primary care support structures (ROS); regional projects such as “Hartslag Limburg”; and local projects of hospitals and community health organisations (GGD-en). Presence and impact of these activities show large regional variation. To enhance national continuous improvement in primary care, the NHG provides a formal accreditation and improvement programme (= the implementation strategy in this project). This programme consists of systematic audit on the basis of validated performance indicators, feedback to practices, improvement plans according to principles of quality management, and follow-up contact on these plans after one year. If the procedure is performed, the practice receives a certificate that provides accreditation for a specified time period. A substantial number of performance indicators is related to CVD. Pilot evaluations by our research centre showed that the procedure was feasible and acceptible for GPs, required substantial time investment, and that plans for improvement were indeed developed by practices.

Effectiveness on implementation strategy
Improvement of professional performance and organisation of care can be expected from the programme, on the basis of its educational components [2-4]. A formal accreditation procedure could be effective [5,6], although evidence was mainly related to hospitals. This evidence is reviewed later in this project description.
In recent years much has been written on performance indicators, accreditation, pay-for-performance, and public reporting [7]. These new, promising approaches use of market forces and pressure for accountability, but evidence on effect and efficiency is very limited [6-8]. For instance, accreditation linked with pay-for-performance might have improved British general practice for chronic diseases, but it also implied high cost [9].
It is therefore important to learn more about the effectiveness and efficiency of the formal accreditation and improvement procedure. Not only for general practice, but also for other health care sectors. Firstly, the added value of formal accreditation remains unclear as most studies on accreditation have reported associations with quality of care rather than effects. Besides direct educational effects, there may be indirect effects related to preparing for the accreditation. Practice development, particularly the introduction of practice nurses, could be crucially important to organise and provide structured chronic care [10]. Secondly, the efficiency of a formal accreditation procedure is largely unknown. Finally, there might be unintended negative consequences.

**Relevantie / Relevance**

Policy developments.
Many programmes have been set up to improve healthcare for patients with cardiovascular diseases, such as Hartslag Limburg, Vascular Risk Management (Hartstichting), Integrated CVA care, and a national FH programme. The guideline on cardiovascular risk management was published by the NHG late 2005 and a slightly revised version of the NHG guideline in 2006 as a multidisciplinary guideline [1]. Other guidelines on cardiovascular diseases were updated in recent years. The NHG has developed a number of products and activities to implement these guidelines, including a national kick-off conference for GPs (December 2005) and a supportive package (‘kwaliteitskoffer’) used in the accreditation programme consisting of educational materials and software for assessment of cardiovascular risk.

Motivation for this study.
The national programme of accreditation and improvement is an innovative, promising approach to quality improvement in healthcare. The programme is not yet evaluated, so we expect that we can further enhance the impact by rigorous evaluation. Improvement of cardiovascular care remains high on the professional and societal agenda, because of its impact on population health. A focus on cardiovascular diseases is necessary for consideration of health outcomes in the evaluation and for in-depth analysis of determinants of improvement of quality of care. CVD has the advantage of a strong evidence base, which allows robust quantitative estimations of effectiveness and efficiency. The

knowledge generated by this study is also relevant outside general practice and cardiovascular treatment. Scientific knowledge of the effect and efficiency of accreditation is scarce, but highly relevant as accreditation is based on new theoretical models for quality improvement (new to health care at least). There is a time window of opportunity for rigorous evaluation of accreditation in general practice, because not all general practices have yet gone through the accreditation process.

Patient population.
The total prevalence of coronary heart diseases (CHD) is 51 and 33 per 1000, respectively, in men and women. The prevalence of cerebrovascular attacks (CVA) is 12 per 1000, both in men and women.These prevalence increase with age. In adults aged 60-64 years, for instance, the prevalence of CHD is 152 and 55, respectively, in men and women. At age 60-64 years the prevalence of CVA is 29 and 17, resp, in men and women. The prevalence of CVD is higher in specific ethnic groups.
The total number of patients with CVD in an average general practice is estimated on 100-150; most are 55 years or older. All figures were derived from note 1 in the guideline [1].

Usual care.
Recent data from representative samples of general practice on usual cardiovascular treatment of patients with CVD are not available. Data from the National Study in a nationally representative network of about 100 general practices in 2002 provides some figures [11]. The overall CVD risk was documented in 57% of patients with diagnosed CVD. In patients who received antihypertensives 33% had received diuretics (first choice medication). A total of 66% of the patients with hypercholesterolemia used statins. Use of aspirine in patients with AP, TIA or PAD varied from 34% (PAD) to 72% (TIA). Another study in a large number of practices showed that GPs gave advice as recommended in 60 to 90% of the consultations with CVD patients [9]. Improvement of cardiovascular care in general practice in the Netherlands has proven to be possible [9,10]. Usual care is unlikely to meet the new treatment targets. Patient adherence and patient perspectives are not examined in this study.

Potential for improvement.
The potential effect on health depends on the effectiveness of treatment (pharmacological and life-style advice) and on the discrepancies between current practice and recommended treatment. Treatment can reduce the estimated risk of cardiovascular mortality by 20% or more (relative risk reduction) [1]. We can safely assume that a substantial proportion of the patients with CVD does not receive optimal treatment. We expect that accreditation results in an increase of + 10% of patients, who have hypertension and cholesterol levels below cut off points as recommended in the guidelines [2]. If these patients had 10 year mortality risk of 50%, this would imply 10 saved lives per 1000 patients with CVD over a period of 10 years. The guideline on cardiovascular risk management explicitly considered costs and the recommendations therefore reflect consensus on what is acceptable efficiency [1]. However, costs of the implementation were not included in these considerations, although the added cost of implementation (e.g. GP time spent on education, practice management, or improvement projects) can be substantial. E.g. a price of 5538 euro per 3 years has been set for the accreditation of a reference general practice (normpraktijk)(in 2006)- excluding practice visitor costs.

**Kennisoverdracht, implementatie, bestendiging / Knowledge transfer, implementation, consolidation**

AIMS
The knowledge transfer related to this project aims firstly to support the national programme for accreditation and improvement of general practice, particularly regarding improvement of cardiovascular risk management. Secondly, it aims to enhance linkages with other parties and initiatives in cardiovascular care in The Netherlands. Thirdly, it aims to contribute to the knowledge on institutional accreditation in general, not just in primary care of family practice but also in other healthcare sectors.

STAKEHOLDERS
These include general practitioners, practice nurses (POH), specialised CVD nurses, practice assistants, and accreditation workers who visit the practices. A wide range of other providers is involved in cardiovascular care, such as internists, cardiologists and neurologists. Other stakeholders are hospitals, health insurers, regional support structures (ROS), and national policy makers.

CONTEXT
Improving cardiovascular risk management is influenced by a range of improvement programmes and factors in patients, providers, and systems. The national programme for practice accreditation in general practice addresses one important provider of cardiovascular care: the primary care practice. The NHG accreditation procedure is an established procedure, for which practices pay a specified prices that is partly reimbursed by Dutch health insurers. The recommendations for cardiovascular care underlying the accreditation and improvement are shared by all health professions. Other initiatives to improve general practice, such as the national FH programme or projects by ROS, could well link to the practice accreditation. For instance, it could help practices to improve in a specific domain. Accreditation of integrated care programmes, covering various care providers (starting with diabetes mellitus), is foreseen in the near future. This accreditation will presume accreditation of participating general practices.

PLANNED ACTIVITIES
The plans for knowledge transfer have been elaborated for each of the specific objectives:

1. Support to national accreditation and improvement programme in general practice

A representative of the Dutch College of GPs (NHG) is co-project leader and responsible for the improvement programme. The proposed project will start in 2008 and provide results in 2010. Project results will be presented to representatives of the professional bodies (LHV, NHG) and a paper for a national journal (e.g. Huisarts en Wetenschap) will be written to inform a wider audience of GPs. The board of the NHG has a strong intention to use the project results for improving the content and methods of the accreditation programme.

2. Enhancing linkages to other parties and initiatives in cardiovascular care.

General practice is one of the several health providers of cardiovascular care; integration of the various types of cardiovascular care is crucial. The NHG quality improvement activities are very well linked to other parties and initiatives on cardiovascular disease in Dutch health care. The guideline on cardiovascular risk management (NHG/CBO) was developed in a collaboration of all relevant health professionals, including GPs, internists, cardiologists, neurologists, etc.. Specific shared care guidelines (LTA's) were developed by NHG with neurologists (on TIA/CVA) and with cardiologists (on MI aftercare). NHG is currently developing a new programme on primary prevention of cardiovascular diseases, which involves (among others) community health organisations (GGD-en) and the Dutch Heart Foundation. Further enhancement of these linkages across disciplines and sectors is an important objective of this project. Patients organisations (NP/CF, CG-Radd, St. Hoofd, Hart en Vaten), Netherlands Heart Foundation, health insures (ZN), Ministery of Health will be informed at an early stage about project. Results will be translated into recommendations for policies and contracting. A special link is sought with the EPA Cardio project (2006-2009), which is an European project on improving cardiovascular care in general practice in 10 countries, led by the project leader.

3. Dissemination of knowledge on effectiveness and efficiency of accreditation

It has already been noted that the scientific knowledge on the effectiveness of institutional accreditation is limited [5,6]. Most evidence is from observational studies in U.S. hospitals, which did not consider costs. The results of this project are innovative for a number of reasons: it relates to office-based primary care practices outside the United States; it is based on a prospectively controlled study; and it considers efficiency of the accreditation, that is the ratio of invested cost and benefits.
Policy makers in other healthcare sectors, who have an interest in accreditation/certification will be identified . We will send them a summary of the project and its results. The (inter) national scientific community will be informed with scientific papers and presentation at scientific conferences.

**Doelstelling / Objective**

The aim of the study is to determine the effectiveness and efficiency of the national programme for accreditation and improvement in primary care, focused on patients with established cardiovascular diseases (secondary prevention). Accreditation is a procedure that is already provided, not just in general practice but in many health care sectors. Given the high expectations and investments into it, evaluation of its effectiveness and efficiency is highly needed. Practice accreditation is interesting because it partly uses new principles to induce improvement, such as market forces and pressure for accountability. Evidence on its impact is as yet limited, particularly in office-based primary care practices.

The specific research questions are:

1. What is the effectiveness of the accreditation and improvement programme on compliance with performance indicators for cardiovascular risk management compared to usual care?

2. What is the effectiveness of this programme, after taking into account the reported exposure to other activities to improve cardiovascular care and other potential confounders?

3. What is the effectiveness of this programme on attainment of practice-defined goals and what are its perceived unintended consequences?

4. What is the efficiency of the programme compared to usual care in the observed period regarding the primary outcomes?

5. What is the estimated efficiency of the programme compared to usual a certain period of 10 years regarding primary outcomes?

**Plan van aanpak / Strategy**

STUDY DESIGN
Randomised controlled trial (RCT) with a block design. This design implies that random allocation is focused on clusters of activities, so that each study group is intervention condition for one domain of activities and control condition for another domain of activities. General practices are recruited from practices that wish to start the accreditation procedure. Practices allocated to the intervention group will be requested to focus their first improvement plans on cardiovascular diseases. Practices allocated to the control group will be asked to focus their first improvement plans on other domains than cardiovascular disease (they may target CVD later). The evaluation compares cardiovascular performance indicators between both groups at follow up (=T1), considering baseline scores (=T0). The control group improvement activities are not evaluated, as these are heterogeneous.

Many good evaluations of quality improvement interventions (also in high impact journals) were RCTs with a block design. This design has the obvious advantages of randomisation, but it assumes that improvement activities in the control group do not influence cardiovascular indicators. It is indeed

unlikely that audit and feedback alone (i.e. on cardiovascular care in the control group) has such impact [2]. To further reduce the potential chance for bias we will ask control group practices to avoid diabetes mellitus in their first improvement activities as this may overlap with CVD.
We have considered the possibility of a waiting list control group of practices. This was considered not feasible by those responsible for the practice accreditation programme, both for the practices (most of which have done efforts to prepare for the accreditation) and for the accreditation organisation itself (there is currently no waiting list).

RANDOMISATION PROCEDURE
A computer generated list of random numbers is used to randomly allocate practices to equilly sized intervention group or control group. This will be done in proportions of the total sample, which is recruited in a 12 month inclusion period.

PRACTICE POPULATION
In an inclusion period of 12 months general practices, which volunteer for accreditation, will be invited to participate in this evaluation. The sample is composed of volunteers for accreditation and therefore not nationally representative for general practices in the country. This reflects current practice, in which practice accreditation is a voluntary activity. It implies that study results cannot be generalised to the (currently hypothetical) situation of obligatory accreditation.
Since both study groups (intervention and control groups) start with accreditation, this project cannot pick up non-specific effects of the accreditation and improvement programme. For instance, we expect that practices prepare for accreditation by improving their practice (e.g. involve a practice nurse). We intend to compare the groups with other, independent samples of practices which provide data on cardiovascular care to get an impression of the representativeness of our sample of practices. In particular, we will use data from a sample of 36 practices that is recruited for an international observation study on cardiovascular risk management in 10 countries in the years 2007 - 2008, led by by the applicant.

PATIENT POPULATION
All data on patients refer to patients with established cardiovascular diseases. Following the guideline on cardiovascular risk management [1], the focus is on atherosclosis-based diseases such as myocardial infarction (MI), angina pectoris (AP), transient ischaemic attack (TIA), cerebrovascular attack (CVA), aneurisma aorta, and peripheral arterial disease (PAD). Diagnoses are based on medical records in general practice; attempts to verify the diagnoses (for instance, against recommendations in guidelines) are beyond the scope of this study. Most performance indicators relate to the total number of patients with CVD in the practice; some measures relate to subgroups within this total group, such as the smokers or those with systolic blood pressure measurements. Patients are treated by the GP, although they be simultaniously treated by medical specialists. Separate patient samples are taken at baseline and at follow-up, although individual patients might be in both samples. The procedures of the chart audit in the accreditation procedure are followed in this study. This implies that each practice provides a random (or systematic) sample of 30 patients per indicator (for a number of indicators the same sample per practice is used).

INTERVENTION GROUP
The intervention starts with the standard accreditation and improvement procedure, which will be elaborated below. For the purpose of this study practices in the intervention group are requested to focus their first improvement plans on cardiovascular diseases.

The accreditation programme is an existing procedure, since 2006 provided by an indepedendent body (NPA) which has a license to use the accreditation procedure developed by the NHG. The NHG remains

responsible for the content and further development of the procedure; it will be responsible for adequate delivery of the improvement programme in this study. The standard accreditation procedure comprises, firstly, of a comprehensive audit (using validated performance indicators) and written feedback to the practice, which covers a range of clinical domains (mainly chronic diseases), practice management, and patient experiences. A second, obligatory component is the planning of improvements in the practice according to the principles of quality management. The practice team is supported by a trained non-physician consultant. Practices which perform the procedure as planned are all accredited, so accreditation does not imply that a certain minimum score has been obtained (the latter is usually labelled certification). Uncertainty about the added value of this last component for quality improvement is a major motivation for this project proposal. In the pilot study of the accreditation program by our centre in 30 practices all succesfully managed to develop improvement plans, so the procedure itself was feasible and promising (Braspenning, Bouma, Witmer, personal communication).

For the purpose of this study we will ask practices in the intervention group to focus their first improvement plans on cardiovascular diseases. Otherwise we would evaluate practices in domains, which have not been targetted by them in their improvement plans. They will, however, received feedback on CVD indicators as this cannot be organised differently and has probably little impact by itself. More specifically, we will ask to set targets related to process and outcomes of cardiovascular care (not only improvement of registration of cardiovascular disease in the medical record system). We will take care that practices receive a minimum of 4 hours support by outreach consultants, which is available in all regions. Also, we will provide the practices with examples of improvement plans, which saves time and would make study participation attractive.

CONTROL GROUP
The control group also starts with the standard accreditation and improvement procedure, which has been described above. For the purpose of this study, they will be asked to focus their first improvement plans on other domains than cardiovascular disease or diabetes mellitus. They are allowed, however, to improve their registration of chronic diseases.

PRIMARY AND SECONDARY OUTCOMES
The audit in the accreditation and improvement programme includes 11 indicators for clinical performance and outcomes and 6 indicators for practice management relevant for cardiovascular risk management. These indicators were developed according to systematic procedures and have been reasonably validated [11]. This study uses all these indicators as outcomes, measured at baseline (as part of the standard accrediation procedure) and at follow-up (=18 months later). The measures refer to, for instance, recording of cardiovascular risk factors in the last 15 months, provision of cholesterol lowering medication (e.g. statins), and availability of systems for labelling and recalling patients. These clinical and organisational indicators are secondary outcomes, except for the following three that we selected as primary outcomes:
1. The percentage of patients in the practice with known established CVD who have systolic blood pressure below 140 mmHG.
2. The percentage of patients in the practice with known established CVD who have LDL cholesterol below 2,5 mmol/l.
3. The percentage of patienst in the practice with known establsihed CVD with a record that aspirin, an alternative anti-platelet therapy or an anti coagulant has been prescribed.

The baseline measurement of these outcomes is based on the audit which is part of the accreditation procedure. Data refer to aggregated figures on practices. The follow-up measurement is performed by us according to the same procedure, after informed consent by patients, focusing on these 17 indicators (not the complete accreditation). The follow-up data will be available at patient level, so that linkage to

other measures (resource use, patient characteristics) can be made at patient level rather than at practice level.

OTHER MEASURES
Other measures in the evaluation have been divided into five categories :

1. Practice goals for improvement and goal attainment.
At follow up T1 the practice teams in both groups will report on the chosen goals for improvement at baseline (as part of the accreditation procedure) and on perceieved achievements on these goals (as well as other perceived achievements) at follow-up. These specific goals are likely to vary across practices, but they reflect closest the chosen goals for improvement. We will use a simple "likert" type question format (agree-disagree) for the goal attainment at follow-up.

2. Resource use for the economic evaluation.
This measurement focuses on the patients sampled for measuring the primary outcomes. At follow up T1 resource use is measured in short observation periods, and then linear extrapolated to the full 18 months observation period [21]. Items of use of healthcare will be extracted from the medical records with a retrospective 3 month observation period. These items include number of contacts in the practice (face to face, telephone, email), use of various types of cardiovascular medication, use of hospital care or other care providers for cardiovascular diagnosis or therapy. Additional information will be collected with patient questionnaires, particularly on other healthcare use (e.g. home care) and productivity losses, using a 2 weeks retrospective observation period. Also, short questionnaires at follow-up (T1) are used in both groups to document time and other resources of practice teams spent on quality improvement in the total period of 18 months, including the accreditation procedure (in the intervention group only) and other relevant quality improvement.

3. Exposure to other quality improvement activities.
At follow-up T1 a brief questionnaire will be completed by the GPs in both study groups to report on their exposure to relevant professional education and practice improvement activities (e.g. ROS, PAOG, training for practice nurses, etc.). A list of such activies will be defined in consultation with various parties (eg. Heart Foundation, Primary Care Supports structures-ROS, etc.) and a simple yes/no answering format is applied to each activity.

4. Potential confounders
At follow up potential confounders will be measured. These include patient characteristics, particularly patient age, gender and multimorbidity. Data on these factors will be added to the chart audit in 30 patients with CVD in each practice. Furthermore, we collect data on practice characteristics that may influence the effectiveness and efficiency of the implementation strategy. These include practice size (number of registered patients), physician workload (hours per week), volume of assistance in the practice, delegation of medical tasks to assistants, and involvement of practice nurses in chronic care. These practice characteristics have shown to be associated with better chronic disease management in Dutch general practices [12]

5. Process evaluation. Semi-structured telephone interviews with GPs in the internvention group are done to assess their experiences with the accreditation procedure. Rather than examining general barriers for change we focus on possible unintended consequences of the accreditation procedure (positive or negative). These include, among others, tunnel vision (focus on phenomena quantified in the audit), misinterpretation (misleading inferences from performance data), gaming (deliberate manipulation of behaviour to secure strategic advantage) [13]. Positive effects may be a generalised improvement, which is broader than cardiovascular care, and improvement of staff morale.

STUDY POWER
In the practices in the accreditation procedure up to 2006 (n=139) the following median values at practice level were found on indicators referring to patients with CVD: 53% for acceptable blood pressure levels; 36% for acceptable cholesterol levels; and 38% for use of anticoagulents (unpublished data at WOK). These data suggest that the current scores on the primary outcomes are in the range of 35 to 55%, which imply that substantial improvement is possible in many practices. The proposed study has been powered to detect a difference between 55% and 65%.
We expected that the accreditation and improvement programme has an effect of 10% absolute change, which is the median value of effect sizes in a comprehensive review of 235 studies on quality improvement [2]. Other assumptions were a power=0.80, alpha=0.05, and ICC=0.05. Given the sample of 30 patients per practice per indicator, we needed 31 practices in each group. Allowing for drop-out, we aim at 35 practices in each group (n=70 practices in total). This number is feasible, given the recruitment rate for the accreditation in 2006.

DATA-ANALYSIS
Question 1. The primary data analysis is as follows. Firstly, as the study is relatively small, the study groups will first be compared at baseline regarding to known determinants of cardiovascular care and its improvement. This comparison includes patient factors (e.g. age, multimorbidity, etnicity at practice level) and practice characteristics (e.g. availability of nurses, delegation of medical tasks to assistance, practice size). Only factors emerging from previous research are considered to avoid over-correction in the primary analysis. Then the focus will turn to the primary outcomes. A logistic regression model will be constructed for each outcome to analyse these outcomes in relation to group (study, control) and moment (baseline, follow-up). Identified differences between the groups at baseline will be included in this analysis. Random coefficients are included to allow for the clustering of data within practices. Each of the secondary outcomes (clinical and organisational indicators) will be analysed in the same way. Finally, if an internally consistent scale can be constructed (reflected by high reliability coefficients of the combined score), we will develop an aggregated measure of outcome and use this in an similar random coefficients linear regression analysis.

Question 2. Firstly, an aggregated measure will be developed for exposure to relevant other quality improvement activities, if this is internally consistent. Then this measure(s) is added to each of the regression models described above in order to correct for the impact of this exposure in the analysis of effectiveness.

Question 3. A descriptive analyis is performed aimed at determining what proportion of self-defined goals for improvement were achieved by the practices and straightforward listing of the GP views on unintended consequences of the accreditation procedure.

Questions 4 and 5 are dealt with in the economic evaluation.

ECONOMIC EVALUATION

Objectives. The economic evaluation aims to determine the efficiency of the accreditation procedure, taking a societal perspective and two time horizons into account: one containing the observed period (using observed cost and outcomes) (Question 4) and one containing a hypothetical 10 years period (using modelling) (Question 5). The economic evaluation also investigates the potential relationship between costs, performance indicators, and accreditation. The short term economic evaluation provides incremental cost-effectiveness ratio's:
- Incremental cost per percentage patients gained with systolic blood pressure below 140 mmHg

- Incremental cost per percentage patients gained with LDL cholesterol < 2,5 mmol/l.
- Incremental cost per percentage patient gained withaspirin, an alternative anti-platelet therapy or an anti coagulant.

Methods. Data-collection on resource use and performance indicators has been described above. The economic evaluation focuses on the primary outcomes only.

Data-analysis, short term economic evaluation. Costs analyses will be based on the competing health production processes respectively including and excluding resources attributed to accreditation. Specific unit-costs include for example medical care (contacts in general practice, tests, treatments, etc.) and improvement related costs (accreditation tariff, time for audit, planning and implementing improvement, exposure to other relevant quality improvement, etc.). Units of resources are monetary valued on the basis of prevailing Dutch guidelines [22] or national CVZ tariffs. The analysis aims to provide incremental cost effectiveness ratio's (ICERs). The ICERs will be computed and uncertainty will be determined using the bootstrap method or Fieller method. For the short term economic evaluation acceptability curves will be derived that are able to evaluate efficiency by using different thresholds for the ICER (varying the WTP for a precentage patients gained for each of the primary outcomes). Uncertainty in deterministic parameters will be examined with  sensitivity analysis based on the range of extremes.

Data-analysis, long term economic evaluation. The long term economic evaluation uses the input of the short term economic evaluation to estimate the net-benefit of accreditation (expressed in euro saved per patient). The long run economic evaluation will have the characteristics of Markov chain processes with cycle length of one year. The input for the model will come from the short term economic evaluation and other published research, particularly on relations between treatment and health outcomes (such as cardiovascular events). We will perform a systematic review of the very large body of research on CVD, focused on modelling studies that are targetted at our primary outcome measures. Extrapolation to final outcomes will be based on epidemiological models most relevant to the Dutch population. We will then build a model based on this literature (if appropriate an adaptation of an existing model), structured as a comparison between the intervention group and control group. Variable costs (costs, effects, transition probabilities) will be extrapolated linearly or according to a functional character of the variable (for example Cox regression, Weibull distribution, hazard function). Utilities will be assigned to states and collected from literature [23]. The model will be probabilistically analyzed (second order Monte Carlo analysis). Final outcome measures of the model will provide in a net-benefit graph and surrounding confidence intervals. Discounting (at several rates varying between 0% and 5%) will be accounted for. A budget impact analysis will be performed to determine the impact on a national level. Sensitivity analyses will be performed to examine the impact of crucial model inputs and assumptions on net-benefits.

PLANNING
Month 1-12: Practices are included in the project and go through the accreditation procedure.
Month 3-27: Practices work on improving their management of CVD.
Month 18-30: Follow-up measurement in intervention and control practices.
Month 31-36. Data-analysis and reports.

EVIDENCE ON EFFECTIVENESS OF THE RECOMMENDATIONS FOR CARDIOVASCULAR CARE

For a detailed description of this evidence we refer to the clinical guidelines on cardiovascular disease (particularly the notes), which have been recently updated [1]. In summary, life style education and

pharmacological treatment can reduce CVD-related mortality and morbidity 20% or more in 10 years. The absolute risk reduction depends on the absolute risk without treatment, which is usually in the range of 20% to 50% in patients with CVD.

EVIDENCE ON EFFECTVIENESS OF THE NATIONAL PROGRAMME FOR ACCREDITATION AND IMPROVEMENT OF GENERAL PRACTICE

1. Evidence on the activities in NHG practice accreditation
NHG practice accreditation comprises of a set of implementation interventions, including: audit and feedback, outreach visits by trained facilitators, and planning improvements according to the quality management principles. This multifaceted implementation has shown to be effective for improving general practice care in the Netherlands in at least two randomized trials, both of which were performed by our research group [3,4,14]. This result is consistent with the wider international literature on combinations of audit and feedback with professional education for improving preventive care [15].
a. A cluster randomised trial aimed to evaluate the effects of feedback reports combined with outreach visits from trained non-physicians on the clinical decision making of 185 GPs (124 practices) in cardiovascular care [3,4] . The evaluation relied on prospective recording of patient encounters by the participating GPs (30 101 clinical decisions at baseline and 22 454 decisions post intervention). A significant improvement was found for 5 of the 12 indicators: assessment of risk factors in patients with hypercholesterolaemia (OR 2.04) or angina pectoris (3.07), provision of information and advice to patients with hypercholesterolaemia (1.58) or hypertension (1.58), and checking for clinical signs of deterioration in patients with heart failure (4.11).
b. A cluster randomised trial in 49 general practices aimed to study the effects of a team-based model for continuous quality improvement on primary care management [14]. In the intervention group, a facilitator helped the teams to select suitable topics for quality improvement and follwed a structured approach to achieve improvement objectives (five meetings per practice). A significant intervention effect was found for the number of improvement objectives actually defined and succesfully completed. There was a non-significant trend that intervention practices improved on aspects of practice management.

2. Evidence on the effectiveness of the accreditation procedure
A systematic review of the literature on regulatory interventions in health systems provided evidence on the effect of institutional accreditation and professional certification (and 8 other regulatory interventions) [5,6].

Methodology: Studies were searched in Medline, Cochrane Database, DARE, King's Fund Library, and a number of grey literature sources (final searches run in July 2006). Specific search terms have been reported in the appendix and include among others: accredit*, revalid*, certif*, visitatie (p.111). A wide range of research designs was included, covering both experimental and observational evaluations. A total of 1400 Medline abstracts on institutional regulation and 1319 abstracts on professional regulation were checked. Study results were reported descriptively only.

Results. Institutional accreditation was defined as “a formal process by which an external body, usually a non-governmental organisation, assesses whether a healthcare organisation meets predetermined and published standards.” (p38) A total of 14 evaluations were found, mainly observational studies in U.S. hospitals, which “showed some evidence for an association between quality of care and accreditation status”. This is no evidence for causality, because “the association could be explained by high performing organisations chosing to participate in accreditation”. (p42) No summary measures for size of effect were given.
Professional certification was defined as “an acknowledgement of a pre-determined level of achievement

of performance, generally recognising achievements exceeding those set as minimum acceptable standards"(p58) A review of studies up to 1999 was found [16], which included 13 studies. An additional 9 studies were found (total n=22 studies). All studies had observational designs and many had methodological limitations. The reviewers report that "s substantial amount of evidence links certification with improved quality of care." (p.65) No summary measures for size of effect were given. Studies which included family physicians showed that board certification was associated with greater compliance with guidelines in treatment of acute myocardial infarction (AMI) in the U.S. [17]; and with 15% lower mortality in another study, which acocunted for case mix differences [18]; with better quality of care for 5 out of 34 performance indicators in Australia [19]; better screening, continuity of care, and presribing in Canada [20].

HTA METHODOLOGY STUDY
The following HTA methodology study has been attached to this application: "The long run - short run efficiency paradox in health care". Dossiernr. 80 - 00702-98-009. Aanvraagnr. 9218.

BUDGET AND CO-FINANCING
Practices who wish to start the accreditation procedure pay a fixed price, which is dependend on the practice size and which is (partly) reimbursed by health insurers. This price is paid to NPA (=NHG Practice Accreditation), a independent organisation (BV). In this project 70 practices pay (at least) 3000 euro each (this has been included in the financial supplements in this application). The project team does not control on this budget (it is paid to the NPA organisation), but it is spent on the implementation strategy that is evaluated. A thorough check of the components of the budget in the pre-proposal has led to higher estimations of assistance and material costs; therefore the total budget is higher than in the pre-application.

**Expertise, voorgaande activiteiten en producten / Expertise, prior activities and products**

The Centre for Quality of Care Research (WOK) is a collaboration between the Universities of Maastricht and Nijmegen, part of the KNAW-acknowledged research school CaRE, and in Nijmegen part of the certified Nijmegen Centre for Evidence-Based Practice (NCEBP). The centre is a multidisciplinary group, which includes physicians, nurses, pharmacists, allied health professionals, health scientists, social scientists etc.. In Nijmegen, the output comprised 30 Ph.D. theses and over 200 international publications since 2000, many educational tools and policy reports. The Centre has a close collaboration with many organisations in the Netherlands (NHG, CBO, Trimbos, universities, etc.) and internationally(universities of Heidelberg, Newcastle, Manchester, Cardiff etc.).

The proposed project will be situated in the WOK programme on implementation of evidence-based practice. Particularly relevant are the previous and ongoing studies on cardiovascular diseases in this programme. Previous studies included implementation trials of outreach visits and feedback (Ph.D.s Hulscher, Frijling, Lobo), education on cholesterol management (Ph.D. Vd Weijden), diabetes care (Ph.D. Dijkstra). Currently ongoing Ph.D. studies related to cardiovascular diseases include:
- implementation trial of risk communication by practice nurses (ZonMW, DO)
- implementation trial of motivational counseling in diabetes patients (ZonMW, Prev)
- observational study on chronic heart failure in general practice (ZonMW, DO)
- pilot study on improving chronic heart failure management in general practice (CZ and VGZ insurers)
- international comparitive study of cardiovascular risk management in 10 countries, led by WOK (German funder)

The Dutch College of General Practitioners (NHG) has currently two main divisions: (a) a department for guideline development and science, which has developed over 80 clinical practice guidelines, which are well received by GPs, (b) a department for implementation and quality improvement, which has developed a wide range of materials for continuing education, patiënt education, and software. The NHG

lead the programme on accreditation and has invested substantial amounts of time, budget and expertise in this programme.

**Publicaties / Publications**

Examples from a much longer list of publications are provided (>300 papers)

CVD AND DIABETES

Meulepas MA, Braspenning JC, de Grauw WJ, Lucas AE, Harms L, Akkermans RP, Grol RP. Logistic support service improves processes and outcomes of diabetes care in general practice. Fam Pract. 2006 Nov 1; [Epub ahead of print]

Dijkstra RF, Niessen LW, Braspenning JC, Adang E, Grol RT. Patient-centred and professional-directed implementation strategies for diabetes guidelines: a cluster-randomized trial-based cost-effectiveness analysis. Diabet Med. 2006 Feb;23(2):164-70.

Dijkstra RF, Braspenning JC, Huijsmans Z, Akkermans RP, van Ballegooie E, ten Have P, Casparie T, Grol RP. Introduction of diabetes passports involving both patients and professionals to improve hospital outpatient diabetes care. Diabetes Res Clin Pract. 2005 May;68(2):126-34. Epub 2005 Jan 11.

van Steenkiste B, van der Weijden T, Timmermans D, Vaes J, Stoffers J, Grol R. Patients' ideas, fears and expectations of their coronary risk: barriers for primary prevention. Patient Educ Couns. 2004 Nov;55(2):301-7.

van Steenkiste B, van der Weijden T, Stoffers HE, Grol R. Barriers to implementing cardiovascular risk tables in routine general practice. Scand J Prim Health Care. 2004 Mar;22(1):32-7.

Frijling B, Hulscher ME, van Leest LA, Braspenning JC, van den Hoogen H, Drenthen AJ, Grol RP. Multifaceted support to improve preventive cardiovascular care: a nationwide, controlled trial in general practice. Br J Gen Pract. 2003 Dec;53(497):934-41.

Frijling BD, Lobo CM, Keus IM, Jenks KM, Akkermans RP, Hulscher ME, Prins A, van der Wouden JC, Grol RP. Perceptions of cardiovascular risk among patients with hypertension or diabetes. Patient Educ Couns. 2004 Jan;52(1):47-53.

Frijling BD, Lobo CM, Hulscher ME, Akkermans RP, van Drenth BB, Prins A, van der Wouden JC, Grol RP. Intensive support to improve clinical decision making in cardiovascular care: a randomised controlled trial in general practice. Qual Saf Health Care. 2003 Jun;12(3):181-7.

Lobo CM, Frijling BD, Hulscher ME, Bernsen RM, Braspenning JC, Grol RP, Prins A, van der Wouden JC. Improving quality of organizing cardiovascular preventive care in general practice by outreach visitors: a randomized controlled trial. Prev Med. 2002 Nov;35(5):422-9.

Frijling BD, Lobo CM, Hulscher ME, van Drenth BB, Braspenning JC, Prins A, van der Wouden JC, Grol RP. Provision of information and advice in cardiovascular care: clinical performance of general practitioners. Patient Educ Couns. 2002 Oct -Nov;48(2):131-7.

Frijling BD, Lobo CM, Hulscher ME, Akkermans RP, Braspenning JC, Prins A, van der Wouden JC, Grol RP. Multifaceted support to improve clinical decision making in diabetes care: a randomized controlled trial in general practice. Diabet Med. 2002 Oct;19(10):836-42.

Beyer M, Gensichen J, Szecsenyi J, Wensing M, Gerlach FM. Wirksamkeit von Disease-Management-Programmen in Deutschland – Probleme der medizinischen Evaluationsforschung anhand eines Studienprotokolls. Zeitschrift für Ärtztliche Fortbildung und Qualität in Gesundheitswesen 2006;100:355-364.

Van der Weijden T, Timmermans DRM, Wensing M. “Dus alles is goed dokter” Hoe informeer ik mijn patiënten over grote en kleine risico’s. Huisarts en Wetenschap 2006;49:550-553.

QUALITY IMPROVEMENT AND IMPLEMENTATION

Grol R, Grimshaw J. From best evidence to best practice: effective implementation of change in pratients’ care. Lancet 2003;362:1225-30.

Grol R, Wensing M. What drives change: barriers and incentives to achieving evidence based practice. Med J Austr 2004;180:S57-60.

Grol R, Bosch M, Hulscher M, Eccles M, Wensing M. Planning and studying improvement in patient care: the use of theoretical perspectives. Milbank Quaterly 2007 (in press)

Bosch M, Van der Weijden T, Wensing M, Grol R. Tailoring quality improvement interventions to identified barriers: a multiple case analysis. J Eval Clin Pract 2006 (in press)
Rhydderch M, Edwards A, Marshall M, Elwyn G, Grol R. Developing a facilitation model to promote organisational development in primary care practices. BMC Fam Pract. 2006 Jun 19;7:38.

Wensing M, Wollersheim H, Grol R. Organizational interventions to implement improvements in patient care: a structured review of reviews. Implement Sci. 2006 Feb 22;1:2.

Dijkstra R, Wensing M, Thomas R, Akkermans R, Braspenning J, Grimshaw J, Grol R. The relationship between organisational characteristics and the effects of clinical guidelines on medical performance in hospitals, a meta-analysis. BMC Health Serv Res. 2006 Apr 28;6:53.

Engels Y, Dautzenberg M, Campbell S, Broge B, Boffin N, Marshall M, Elwyn G, Vodopivec-Jamsek V, Gerlach FM, Samuelson M, Grol R. Testing a European set of indicators for the evaluation of the management of primary care practices. Fam Pract. 2006 Feb;23(1):137-47. Epub 2005 Oct 21.

Wensing M, van den Hombergh P, Akkermans R, van Doremalen J, Grol R. Physician workload in primary care: what is the optimal size of practices? A cross-sectional study.
Health Policy. 2006 Aug;77(3):260-7. Epub 2005 Aug 29.

Rosemann T, Wensing M, Rueter J, Szecsenyi J. Referrals from general practice to consultants in Germany: If the GP is the initiator, patients' experiences are more positive. BMC Health Serv Res 2006;6:5.

Wensing M, Schattenberg G. Response rates after three follow-up procedures in a survey among elderly adults: a randomised trial. J Clin Epidem 2005;58:959-61.

Rhydderch M, Edwards A, Elwyn G, Marshall M, Engels Y, Van den Hombergh P, Grol R. Organizational assessment in general practice: a systematic review and implications for quality

improvement. J Eval Clin Pract. 2005 Aug;11(4):366-78. Review.

Ouwens M, Wollersheim H, Hermens R, Hulscher M, Grol R. Integrated care programmes for chronically ill patients: a review of systematic reviews. Int J Qual Health Care. 2005 Apr;17(2):141-6. Epub 2005 Jan 21.

Anderson P, Kaner E, Wutzke S, Funk M, Heather N, Wensing M, Grol R, Gual A, Pas L; WHO Brief Intervention Study Group. Attitudes and managing alcohol problems in general practice: an interaction analysis based on findings from a WHO collaborative study. Alcohol Alcohol. 2004 Jul-Aug;39(4):351-6.

Laurant MG, Hermens RP, Braspenning JC, Sibbald B, Grol RP. Impact of nurse practitioners on workload of general practitioners: randomised controlled trial. BMJ. 2004 Apr 17;328(7445):927. Epub 2004 Apr 6.

van Eijken M, Tsang S, Wensing M, de Smet PA, Grol RP. Interventions to improve medication compliance in older patients living in the community: a systematic review of the literature. Drugs Aging. 2003;20(3):229-40. Review.

Wensing M, Van der Weijden T. Kwaliteitsverbetering: over heilige huisjes en voortschrijdend inzicht. Huisarts en Wetenschap 2004;47:263-5.

Wetzels RV, Harmsen M, Van Weel C, Grol R, Wensing M. Interventions for improving older patients' involvement in primary care episodes (review). Cochrane Library 2007; issue 1.

Smolders M, Laurant M, Van Wamel A, Grol R, Wensing M. What determines the management of anxiety disorders and its improvement? J Eval Clin Pract 2007 (in press).

Plas M, Fleuren M, Friele R, Haaijer-Ruskamp F, Keijsers J, Ravensbergen J, Klazinga N, Wensing M. Begrippenkader voor het 'wat' en het ´waarom' bij implementatie van vernieuwingen in patiëntenzorg en preventie. Tijdschrift Sociale Gezondheidszorg 2007 (in druk)

**Referenties / References**

1 Nederlands Huisartsen Genootschap. NHG-standaard M84: Cardiovasculair risicomanagement. www.NHG.artsennet.nl (versie 2007).

2 Grimshaw J, Thomas RE, Maclennan G, Fraser C, Ramsay CR, Vale L, Whitty P, Eccles MP, Matowe L, Shirran L, Wensing M, Dijkstra R, Donaldson C. Effectiveness and efficiency of guideline dissemination and implementation trategies. Health Technology Assessment 2004;8(6).

3 Frijling BD, Lobo CM, Hulscher M et al. Multifaceted support to improve preventive cardiovascular care: a nationwide, controlled trial in general practice. Br J Gen Pract 2003;53:934-941.

4 Frijling BD et al. Intensive support to improve clinical decision making in cardiovascular care: a randomised controlled trial in general practice. Qual Saf Health Care 2003;12:181-187.

5 Sutherland K, Leatherman S. Does certification improve medical standards ? BMJ 2006;333:439-441.

6 Sutherland K, Leatherman S. Regulation and quality improvement. A review of the evidence. The Health Foundation, Quest for Quality and Improved Performance, UK, October 2006. (www.health.org.uk/aboutus/publications)

7 Grol R, Grimshaw J. From best evidence to best practice: effective implementation of change in patients' care. Lancet 2003;362:1225-1230.

8 Marhsall MN, Romano P, Davies HTO. How do we maximize the impact of the public reporting of quality of care? Int Qual Health Care 2004;16:S57-63.

9 Roland M. Linking physicians to pay to the quality of care. A major experiment in the United Kingdom. N Engl J Med 2004;351:1448-1454.

10 Laurant M, Reeves D, Hermens R, Braspenning J, Grol R, Sibbald B. Substitution of doctors by nurses in primary care (Review). Cochrane Library, 2004.

11 Van Roosmalen M, Braspenning J, Grol R. Cardiovasculair risicomanagement: praktijkvoering en medisch handelen. In: Braspenning JCC, Schellevis FG, Grol R (red.). Tweede Nationale Studie naar ziekten en verrichtingen in de huisartspraktijk. Kwaliteit huisartsenzorg belicht. Uitgave WOK/Nivel Utrecht/Nijmegen, 2004.

12 Wensing M, Van den Hombergh P, Akkermans R, Van Doremalen J, Grol R. Physician workload in primary care: what is the optimal size of practices? A cross-sectional study. Health Policy 2006;77: 260-267.

13 Freeman T. Using performance indicators to improve health care quality in the public sector: a review of the literature. health Services Management Research 2002;15:126-137.

14 Engels Y, Van den Hombergh P, Mokkink H, et al.. The effects of a team-based continuous quality improvement intervention on the management of primary care: a randomised controlled trial. Br J Gen Pract 2006;56:781-878.

15. Hulscher M, wensing M, Grol R et al. Interventions to improve the delivery of preventive services in primary care. Am J Publ Health 1999;89:737-746.

16 Sharp LK, Bashook PG, Lipsky MSD, Horowitz SD, Miller SH. Specialty board certificiation and clinical outcomes: the missing link. Academic Medicine 2002;77:534-542.

17 Chen J, Rathore SS, Wang Y, Radford MJ, Krumholz HM. Physician Board Certification and the Care and Outcomes of Elderly Patients with Acute Myocardial Infarction. J Gen Intern Med. 2006 Jan 20; [Epub ahead of print]

18 Norcini J, Kimball H, Lipner R Certification and specialization: do they matter in the outcome of acute myocardial infarcrtion. Acad Med 2000;75:1193-1198.

19 Miller G, Britth H, Pan Y, Knox S. Relationship between general practitioner certification and characteristics of care. Med Care 20004;42:770-778.

20 Tamblyn R, Abramowicz M, Daupinee W et al. Association between licensure examination scores and practice in primary care. JAMA 2002;288:3019-3026.

21 Goossens MEJB, Rutten-vanMölken MPMH, Vlaeyen JWS, Van der Linden SMJP. The cost diary: a method to measure direct and indirect costs in cost-effectiveness research. J Clin Epidemiol 2000;53:688-695.

22 Oostenbrink JB, Koopmanschap MA, Rutten FFH. Handleiding voor kostenonderzoek. Methoden en richtlijnprijzen voor economische evaluaties in de gezondheidszorg.

23 Tengs TO, Wallace AMA. One thousand health-related quality-of-life estimates. Medical Care. 2000;38:583-637.

## Financiële gegevens / Financial data

### Geplande duur in maanden / Planned duration in months

36 maanden / months

### ZonMw budget

| | Jaar / Year | | | | | | | | |
|---|---|---|---|---|---|---|---|---|---|
| *Kostenpost / Cost item* | *1* | *2* | *3* | *4* | *5* | *6* | *7* | *8* | *Totaal / Total* |
| Apparatuur | 0 | 0 | 0 | 0 | 0 | 0 | 0 | 0 | 0 |
| Personeel | 86.148 | 92.855 | 99.795 | 0 | 0 | 0 | 0 | 0 | 278.798 |
| Overig | 1.500 | 500 | 3.200 | 0 | 0 | 0 | 0 | 0 | 5.200 |
| Implementatie | 0 | 0 | 0 | 0 | 0 | 0 | 0 | 0 | 0 |
| Materieel | 21.500 | 3.000 | 4.000 | 0 | 0 | 0 | 0 | 0 | 28.500 |
| **Totaal / Total** | **109.148** | **96.355** | **106.995** | **0** | **0** | **0** | **0** | **0** | **312.498** |

### Co-financiering / Cofinancing

| Naam co-financier / Name of cofinancier | Bedrag / Amount | Status |
|---|---|---|
| Nederlands Huisartsen GenootschapImplementatie UTRECHT Nederland | 210.000 | Wordt aangevraagd |

## Bijzondere gegevens / Additional information

### Vergunningen / Permits

| | Vergunning nodig / Permit required? | | Vergunning verkregen / Permit obtained? | |
|---|---|---|---|---|
| | Ja / Yes | Nee / No | Ja / Yes | Nee / No |
| METC/DEC | X | | | X |
| WBO | | X | | X |
| Biohazards | | X | | X |

### Andere vergunningen / Other permits

nvt

**Historie subsidieaanvraag / History grant application**

Deze aanvraag is eerder ingediend bij het programma / This grant application has previously been submitted to the ZonMw programme:

Projectnummer / Project number:

Deze aanvraag is ook bij andere organisaties dan ZonMw ingediend / This grant application has also been submitted to other organizations than ZonMw:

**Ondertekening / Signatures**

| | |
|---|---|
| Naam penvoerder-projectleider:<br><br>M Wensing | Naam bestuurlijk verantwoordelijke:<br><br>D.J. Ruiter |
| Plaats en datum: | Plaats en datum: |
| Handtekening:<br><br>---------------------------------------- | Handtekening:<br><br>---------------------------------------- |

**Application ID:**

**BEGROTINGSOVERZICHT DOELMATIGHEIDSONDERZOEK**

Projectnaam: Accreditering HVZ

| | Jaar 1 | Jaar 2 | Jaar 3 | Totaal |
|---|---|---|---|---|
| | € | € | € | € |
| 1 Personele kosten | 86.148 | 92.855 | 99.795 | 278.798 |
| 2 Materiele kosten | 21.500 | 3.000 | 4.000 | 28.500 |
| 4 Overige kosten | 1.500 | 500 | 212.450 | 214.450 |
| Totale lasten | 109.149 | 96.355 | 316.245 | 521.749 |
| 5 Bijdragen van eigen instelling c.q. derden | - | - | 210.000 | 210.000 |
| **Aangevraagd budget bij ZonMw** | | | | 311.749 |

**Application ID:**

Projectnaam: Accreditering HVZ

1.a PERSONELE KOSTEN JAAR 1:

| nr | Functie | Schaal | Formatie (perc. per jaar) | Bruto salaris (413) | Overhevelings toeslag (4222) | Werkgevers-bijdragen (422) | Subtotaal | Opslag op personeels-kosten 16% | Totaal € |
|---|---|---|---|---|---|---|---|---|---|
| 1 | Projectleider | 14,04 | 0,10 | 6.067 | | 2.245 | 8.312 | 1.330 | 9.642 |
| 2 | HA-Onderzoeker | 12,10 | 0,40 | 23.276 | | 8.612 | 31.888 | 5.102 | 36.990 |
| 3 | Onderzoeksassistentie | 8,10 | 0,50 | 17.650 | | 6.531 | 24.181 | 3.869 | 28.050 |
| 4 | Statistische ondersteuning | 10,12 | 0,10 | 4.387 | | 1.623 | 6.010 | 962 | 6.972 |
| 5 | Secretariaat | 6,10 | 0,10 | 2.829 | | 1.047 | 3.875 | 620 | 4.495 |
| | | | | | | | - | - | - |
| Totaal | | | | 54.209 | | 20.057 | 74.266 | 11.883 | 86.148 |

1.b PERSONELE KOSTEN JAAR 2:

| nr | Functie | Schaal | Formatie (perc. per jaar) | Bruto salaris (413) | Overhevelings toeslag (4222) | Werkgevers-bijdragen (422) | Subtotaal | Opslag op personeels-kosten 16% | Totaal € |
|---|---|---|---|---|---|---|---|---|---|
| 1 | Projectleider | 14,05 | 0,10 | 6.358 | | 2.352 | 8.710 | 1.394 | 10.105 |
| 2 | HA-Onderzoeker | 12,10 | 0,40 | 23.741 | | 8.784 | 32.525 | 5.204 | 37.729 |
| 3 | Onderzoeksassistentie | 8,10 | 0,50 | 18.003 | | 6.661 | 24.664 | 3.946 | 28.610 |
| 4 | Statistische ondersteuning | 10,12 | 0,10 | 4.474 | | 1.656 | 6.130 | 981 | 7.111 |
| 5 | Econ. Evaluator /senior ondz. | 12,10 | 0,05 | 2.967 | | 1.098 | 4.065 | 650 | 4.716 |
| 6 | Secretariaat | 6,10 | 0,10 | 2.885 | | 1.067 | 3.953 | 632 | 4.585 |
| Totaal | | | | 58.428 | | 21.618 | 80.046 | 12.807 | 92.855 |

1.c PERSONELE KOSTEN JAAR 3:

| nr | Functie | Schaal | Formatie (perc. per jaar) | Bruto salaris (413) | Overhevelings toeslag (4222) | Werkgevers-bijdragen (422) | Subtotaal | Opslag op personeels-kosten 16% | Totaal € |
|---|---|---|---|---|---|---|---|---|---|
| 1 | Projectleider | 14,06 | 0,10 | 6.655 | | 2.462 | 9.117 | 1.459 | 10.577 |
| 2 | HA-Onderzoeker | 12,10 | 0,40 | 24.216 | | 8.960 | 33.176 | 5.308 | 38.484 |
| 3 | Onderzoeksassistentie | 8,10 | 0,50 | 18.363 | | 6.794 | 25.157 | 4.025 | 29.182 |
| 4 | Statistische ondersteuning | 10,12 | 0,10 | 4.564 | | 1.689 | 6.253 | 1.000 | 7.253 |
| 5 | Econ. Evaluator /senior ondz. | 12,10 | 0,10 | 6.054 | | 2.240 | 8.294 | 1.327 | 9.622 |
| 6 | Secretariaat | 6,10 | 0,10 | 2.943 | | 1.089 | 4.032 | 645 | 4.677 |
| | | | | | | - | - | - | - |
| | | | | 62.795 | | 23.234 | 86.029 | 13.765 | 99.795 |

**Application ID:**

Projectnaam: Accreditering HVZ

2.a MATERIELE KOSTEN JAAR 1

| Bij verrichting (COTG nr….) | Aantal | Tarief | Totaal |
|---|---|---|---|
| Reiskosten | | | 2.000 |
| Vacatiegelden praktijken | | | 17.500 |
| Congres/scholingskosten | | | 2.000 |
| Totaal | | | 21.500 |

2.b MATERIELE KOSTEN JAAR 2

| Bij verrichting (COTG nr….) | Aantal | Tarief | Totaal |
|---|---|---|---|
| Reiskosten | | | 1.000 |
| Vacatiegelden praktijken | | | - |
| Congres/scholingskosten | | | 2.000 |
| Totaal | | | 3.000 |

2.c MATERIELE KOSTEN JAAR 3

| Bij verrichting (COTG nr….) | Aantal | Tarief | Totaal |
|---|---|---|---|
| Reiskosten | | | 2.000 |
| Vacatiegelden praktijken | | | - |
| Congres/scholingskosten | | | 2.000 |
| Totaal | | | 4.000 |

**Application ID**:

Projectnaam: Accreditering HVZ

4.a OVERIGE KOSTEN JAAR 1

| Omschrijving (461/462) | | Kosten |
|---|---|---|
| Vragenlijsten/printing/porti/drukkosten etc | | 1500 |
| | | 0 |
| | | 0 |
| | | 0 |
| Totaal | € | 1500 |

4.b OVERIGE KOSTEN JAAR 2

| Omschrijving (461/462) | | Kosten |
|---|---|---|
| Vragenlijsten/printing/porti/drukkosten etc | | 500 |
| | | - |
| | | - |
| | | - |
| Totaal | € | 500 |

4.c OVERIGE KOSTEN JAAR 3

| Omschrijving (461/462) | | Kosten |
|---|---|---|
| Vragenlijsten/printing/porti/drukkosten etc | | 1.000 |
| Accrediteringskosten NHG | | 210.000 |
| Proefschrift/ vertaalkosten artikelen | | 1.200 |
| Accountantskosten | | 250 |
| Totaal | € | 212.450 |

**Application ID:**

Projectnaam: Accreditering HVZ

5.a BIJDRAGEN JAAR 1

| | | |
|---|---|---|
| 5.a.1 | | - |
| | | - |
| | | - |
| 5.a.2 | | - |
| | | - |
| | Totaal | - |

5.b BIJDRAGEN JAAR 2

| | | |
|---|---|---|
| 5.b.1 | | - |
| | | - |
| | | - |
| 5.b.2 | | - |
| | | - |
| | Totaal | - |

5.c BIJDRAGEN JAAR 3

| | | |
|---|---|---|
| 5.b.1 | | - |
| | Bijdragen NHG | 210.000 |
| | | - |
| 5.b.2 | | - |
| | Totaal | 210.000 |

## Flow chart

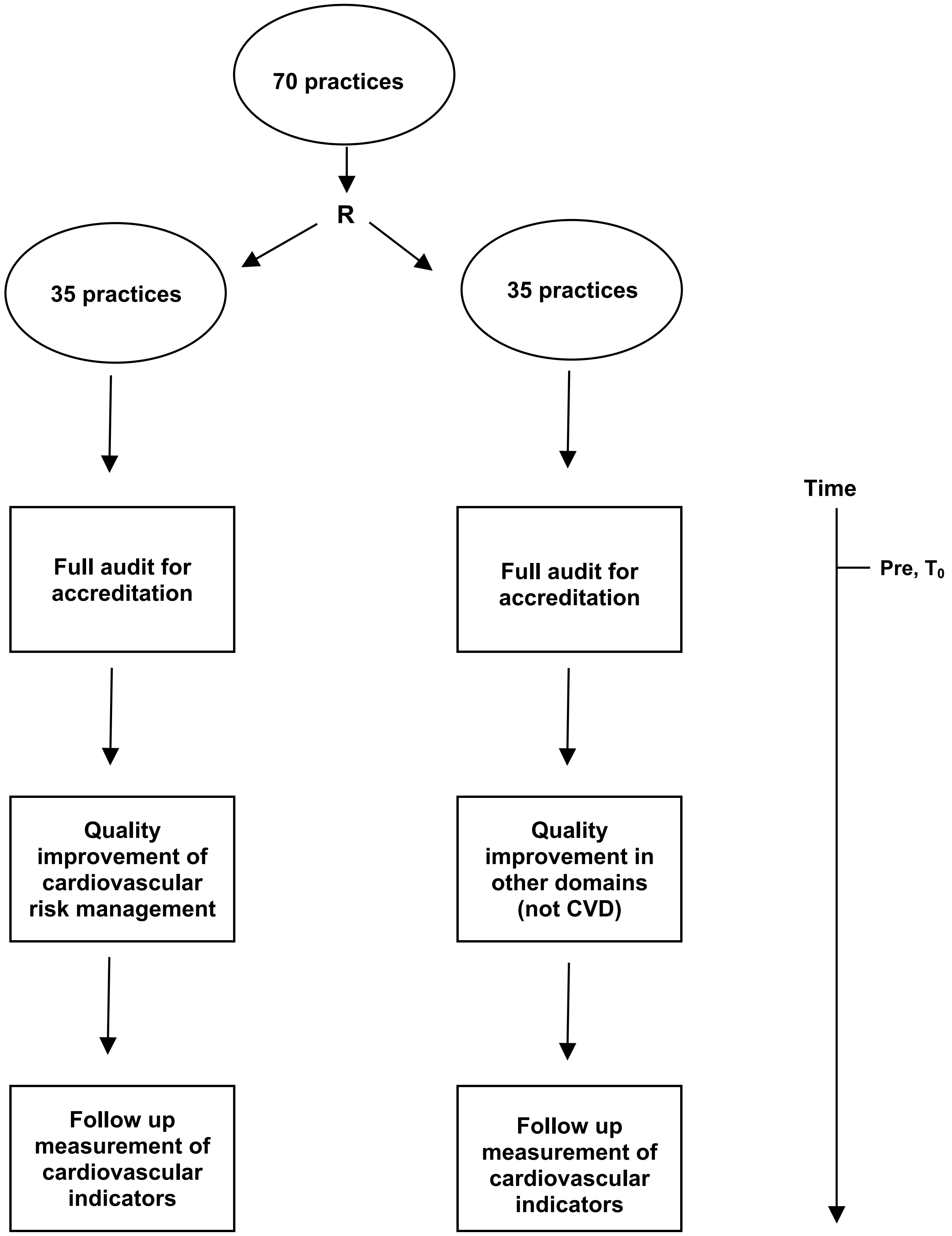
70 practices
R
35 practices
35 practices
Full audit for accreditation
Full audit for accreditation
Quality improvement of cardiovascular risk management
Quality improvement in other domains (not CVD)
Follow up measurement of cardiovascular indicators
Follow up measurement of cardiovascular indicators
Time
Pre, T0

**Informatiebrief voor patiënten (concept versie)**

Informatie voor de deelnemers van dit project van de afdeling Kwaliteit van zorg (WOK) van het UMC St Radboud Nijmegen.

**Geachte heer/mevrouw,**

**Deze brief bevat informatie over een kwaliteitsverbeteringsproject in uw huisartspraktijk.**

**Inleiding**
Jaarlijks worden in Nederland ongeveer 250.000 mensen in het ziekenhuis opgenomen vanwege hart- en vaatziekten, zoals een hartinfarct of een beroerte. Het is mogelijk om uw kans op hart- en vaatziekten kleiner te maken. Een gezonde leefstijl en bepaalde medicijnen kunnen hierbij helpen. Uw huisartspraktijk probeert zo goed mogelijk zorg te geven aan mensen met hart- en vaatziekten.

**Wat is het doel van het onderzoek?**
De huisartspraktijk neemt deel aan een landelijk programma om de huisartsenzorg verder te verbeteren. Het doel van het onderzoek is om te testen of verbeteringen in de huisartspraktijk inderdaad leiden tot betere zorg voor mensen met hart- en vaatziekten. Als individuele patiënt merkt u misschien bepaalde veranderingen in de huisartspraktijk, maar het kan ook zijn dat u persoonlijk niets merkt.

**Hoe ziet het onderzoek eruit?**
Het onderzoek vindt plaats in de huisartspraktijk en wordt uitgevoerd door huisarts en praktijkmedewerkers, ondersteund door een onderzoeksassistent. Op twee momenten worden gegevens verzameld uit medisch dossiers, via vragenlijsten voor patiënten, huisartsen en praktijkmedewerkers. Het onderzoek duurt 18 maanden.

**Wat wordt er van u gevraagd?**
Wij vragen uw toestemming voor het mogen gebruiken van enkele gegevens uit uw medisch dossier voor ons onderzoek. Verder vragen wij u twee keer een schriftelijke vragenlijst in te vullen, die per post wordt gestuurd.

**Wat levert het u op?**
Er is voor u geen persoonlijk voordeel. U helpt de huisartspraktijk om de zorg te verbeteren.

**Zitten er risico's aan het onderzoek?**
U kunt zonder risico deelnemen aan het onderzoek. De Medische Ethische Toetsingscommissie heeft dit onderzoek goedgekeurd. De commissie heeft ontheffing verleend voor de verplichting een verzekering af te sluiten die de door het onderzoek veroorzaakte schade van de proefpersoon dekt. De reden voor deze ontheffing is dat deze commissie van oordeel is dat dit onderzoek naar zijn aard zonder enig risico is.

**Vertrouwelijkheid van de gegevens**
De vragenlijsten kunt u in een antwoordenveloppe aan het UMC St Radboud sturen. U hoeft geen naam en adres in te vullen, die gegevens blijven in de praktijk. U wordt op de universiteit genoteerd met een uniek nummer, en uw gegevens worden dus anoniem verwerkt. Uw naam en adresgegevens op de toestemmingsverklaring gebruiken wij alleen om de vragenlijsten aan u te kunnen versturen.

**Vrijwilligheid van deelname:**
Deelname aan dit onderzoek is geheel vrijwillig en verplicht u tot niets. U kunt zelf beslissen of u aan dit onderzoek deelneemt. Als u wilt deelnemen, vult u de toestemmingsverklaring in en stuurt u deze samen met de eerste vragenlijst in de antwoordenveloppe naar het UMC St Radboud. Of u wel of niet deelneemt, heeft uiteraard geen gevolgen voor de normale zorg die u van uw huisarts ontvangt. U kunt te allen tijde zonder opgaaf van redenen van verdere deelname aan dit onderzoek afzien.

**Vragen?**
Mocht u naar aanleiding van deze informatie of tijdens uw deelname aan het onderzoek of daarna nog vragen of opmerkingen hebben dan kunt u contact opnemen met de hieronder genoemde onderzoeker.

Mede namens uw huisarts,

Jan van Lieshout, huisarts-onderzoeker
Afdeling Kwaliteit van zorg (WOK)
UMC St Radboud
Tel. 024-3615305

**Indicators on cardiovascular risk management in the NHG practice accreditation**

**Clinical indicators and outcomes**

**1** % of patients with CVD* in general practice

| | | | | | |
|---|---|---|---|---|---|
| *Numerator:* | Total number of patients with CVD known in general practice: | | | | |
| *Denominator*: | Total number of patients in practice population: | | | x 100 = | |

* Cardiovascular disease (CVD): Coronary heart disease (angina, myocardial infarct), stroke, transient ischemic attack, aortic aneurism or peripheral vascular disease.

**2** % of patients with CVD in general practice with blood pressure measurement in the past 15 months

| | | | | |
|---|---|---|---|---|
| *Numerator:* | Number of patients with CVD in general practice with systolic blood pressure measurement | | | |
| *Denominator:* | Total number of patients with CVD in general practice | | x 100% = | |

**3** % of patients with CVD in general practice with systolic blood pressure below 140 mmHg

| | | | | |
|---|---|---|---|---|
| *Numerator:* | Number of patients with CVD in general practice in whom the last systolic blood pressure measurement was below 140 mmHg | | x 100% = | |
| *Denominator:* | Number of patients with CVD in general practice with systolic blood pressure measurement | | | |

**4** % of patients with CVD in general practice with LDL cholesterol measurement in the past 15 months

| | | | | |
|---|---|---|---|---|
| *Numerator:* | Number of patients with CVD in general practice with LDL cholesterol measurement | | | |
| *Denominator:* | Total number of patients with CVD in general practice | | x 100% = | |

**5** % of patients with CVD in general practice who are currently treated with cholesterol lowering medication (E.g. statins)

| | | | | |
|---|---|---|---|---|
| *Numerator:* | Number of patients with CVD in general practice who are currently treated with cholesterol lowering medication (E.g. statins) | | x 100% = | |
| *Denominator:* | Total number of patients with CVD in general practice | | | |

**6** % of patients with CVD in general practice with LDL cholesterol below 2,5 mmol/l

| | | | | |
|---|---|---|---|---|
| *Numerator:* | Number of patients with CVD in general practice in whom the last with LDL cholesterol level was below 2,5 mmol/l | | x 100% = | |
| *Denominator:* | Total number of patients with CVD in general practice | | | |

**7** % of patients with CVD in general practice with Body Mass Index recorded in the past 15 months

| | | | | |
|---|---|---|---|---|
| *Numerator:* | Number of patients with CVD in general practice with Body Mass Index recorded | | x 100% = | |
| *Denominator:* | Total number of patients with CVD in general practice | | | |

**8** % of patients with CVD in general practice with a record of smoking status

| | | | | |
|---|---|---|---|---|
| *Numerator:* | Number of patients with CVD in general practice with a record of smoking status | | | |
| *Denominator:* | Total number of patients with CVD in general practice | | x 100% = | |

**9** % of smoking patients with CVD in general practice with a record of non-smoking advice during the past 15 months

| | | | | |
|---|---|---|---|---|
| *Numerator:* | Number of smoking patients with CVD in general practice with a record of non-smoking advice | | | |
| *Denominator:* | Total number of smoking patients with CVD in general practice | | x 100% = | |

10 % of patients with CVD in general practice with a record that aspirin, an alternative anti-platelet therapy or an anti coagulant has been prescribed in the previous year

| | | | | |
|---|---|---|---|---|
| *Numerator:* | Number of patients with CVD in general practice with a record that aspirin, an alternative anti-platelet therapy or an anti coagulant has been prescribed | | x 100% = | |
| *Denominator:* | Total number of patients with CVD in general practice | | | |

11 % of patients with CVD in general practice with blood glucose measurement in the past 5 years

| | | | | |
|---|---|---|---|---|
| *Numerator:* | Number of patients with CVD in general practice with fasting blood glucose measurement | | | |
| *Denominator:* | Total number of patients with CVD in general practice | | x 100% = | |

**Indicators for practice management**

| | | |
|---|---|---|
| 1 | Do you have special consulting hours in your practice for risk management for patients with cardiovascular diseases? | ☐ No<br>☐ Yes |
| 2 | Can you -using your EMD- produce a register of patients with cardiovascular diseases? Meant are patients who had atherotrombotic events: myocardial infarction, angina pectoris, stroke, TIA, aortic aneurism and peripheral vessel disease. | ☐ No<br>☐ Yes<br>☐ Just the following patient groups..… |
| 2 | If yes, how are these cardiovascular disease patients selected? | Selected by<br>☐ Marker<br>☐ ICPC code<br>☐ other: …….. |
| 4 | Do you have a recall system for risk management for patients with cardiovascular diseases? | ☐ No<br>☐ Yes<br>☐ If yes. How is it done? ………………………….. |
| 5 | Do you have task delegation with respect to care for patients with cardiovascular diseases to the practice assistance / POH / practice nurse? | ☐ No<br>☐ Yes<br>☐ If yes. What tasks? |
| 6 | Do you have task delegation with respect to care for patients with cardiovascular diseases to an organization outside your general practice? | ☐ No<br>☐ Yes<br>☐ If yes. What tasks?<br>☐ And where? |
